# Comparison studies identify mesenchymal stromal cells with potent regenerative activity in osteoarthritis treatment

**DOI:** 10.1038/s41536-024-00358-y

**Published:** 2024-04-01

**Authors:** Hongshang Chu, Shaoyang Zhang, Zhenlin Zhang, Hua Yue, Huijuan Liu, Baojie Li, Feng Yin

**Affiliations:** 1 Bio-X Institutes, Key Laboratory for the Genetics of Developmental and Neuropsychiatric Disorders, Ministry of Education, Shanghai Jiao Tong University, Shanghai, 200240 China; 2 Department of Osteoporosis and Bone Diseases, Shanghai Clinical Research Center of Bone Disease, Shanghai Jiao Tong University Affiliated Sixth People’s Hospital, Shanghai, 200233 China; 3 Shanghai Institute of Stem Cell Research and Clinical Translation, Shanghai, 200120 China; 4 Translational Medical Center for Stem Cell Therapy, Shanghai East Hospital, Tongji University, Shanghai, 200120 China; 5 Department of Joint and Sports Medicine, East Hospital, Tongji University School of Medicine, Shanghai, 200092 China

**Keywords:** Mesenchymal stem cells, Stem-cell therapies, Stem-cell research

## Abstract

Osteoarthritis affects 15% of people over 65 years of age. It is characterized by articular cartilage degradation and inflammation, leading to joint pain and disability. Osteoarthritis is incurable and the patients may eventually need joint replacement. An emerging treatment is mesenchymal stromal cells (MSCs), with over two hundred clinical trials being registered. However, the outcomes of these trials have fallen short of the expectation, due to heterogeneity of MSCs and uncertain mechanisms of action. It is generally believed that MSCs exert their function mainly by secreting immunomodulatory and trophic factors. Here we used knee osteoarthritis mouse model to assess the therapeutic effects of MSCs isolated from the white adipose or dermal adipose tissue of *Prrx1-Cre; R26*^*tdTomato*^ mice and *Dermo1-Cre; R26*^*tdTomato*^ mice. We found that the *Prrx1*-lineage MSCs from the white adipose tissues showed the greatest in vitro differentiation potentials among the four MSC groups and single cell profiling showed that the *Prrx1*-lineage MSCs contained more stem cells than the *Dermo1* counterpart. Only the *Prrx1*-lineage cells isolated from white adipose tissues showed long-term therapeutic effectiveness on early-stage osteoarthritis models. Mechanistically, *Prrx1*-lineage MSCs differentiated into Col2^+^ chondrocytes and replaced the damage cartilage, activated Col1 expressing in resident chondrocytes, and inhibited synovial inflammation. Transcriptome analysis showed that the articular chondrocytes derived from injected MSCs expressed immunomodulatory cytokines, trophic factors, and chondrocyte-specific genes. Our study identified a MSC population genetically marked by *Prrx1* that has great multipotentiality and can differentiate into chondrocytes to replace the damaged cartilage.

## Introduction

Osteoarthritis (OA) is a common degenerative disease that affects the knee, hip, and other joints, causing joint pain and disability^1–4^. Genetic makeup, age, physical fitness, and sport injuries are the main causes of OA^2,5,6^. The disease has a significant impact on the quality of life and causes huge socioeconomic burdens, which will become an even greater challenge in the future due to aging of the population^7,8^. At present, OA is treated with conservative and/or surgical approaches, with the former using drugs such as corticosteroids and hyaluronic acid and the latter using cleaning surgery, joint replacement, or chondrocyte implantation^9,10^. The conservative treatment can only temporarily relieve the symptoms while the surgical treatment has a limited use-life^2^. Recently, mesenchymal stromal cells (MSCs) have emerged as a promising treatment for OA^11–14^. More than two hundred related clinical trials have been registered at the clinicaltrials.gov^15^. However, the outcomes have been heterogenous and the effects do not last long^16^. As such, reliable and repeatable MSC product is still in shortage for OA treatment.

MSCs can be conveniently obtained from the bone marrow, adipose tissue (stromal vascular fraction (SVF)), umbilical cord, dermis, or other tissues and they readily expand in vitro^17,18^. They represent a mixture of stromal cells of the above tissues and are defined by the in vitro properties: adherence to culture dishes, expression of CD73, CD90, and CD105 but not CD31 or CD45 (for human MSCs), and capability to differentiate into osteoblasts, chondrocytes, and adipocytes in vitro^19,20^. Preclinical and clinical studies have shown that MSCs have limited ability to differentiate into chondrocytes to regenerate the damaged cartilage^16^. Instead, they exert their function mainly via producing immunomodulatory cytokines, e.g., IDO, TSG6, and PGE2, and trophic growth factors, e.g., HGF, TGFβ, and FGFs, which can act on the immune cells and resident articular chondrocytes, respectively^21,22^. Besides the soluble factors, extracellular vesicles from MSCs can also inhibit inflammation and promote cartilage regeneration^23–25^. These mechanisms of action explain why the in vitro MSC differentiation potential does not reflect their effectiveness in OA treatment^16^.

The heterogeneous nature of MSCs, uncertain mechanisms of action, and lack of markers to predict the therapeutic effects of MSCs constitute the major obstacles of MSC-based therapy^26,27^. In particular, the heterogeneity of MSCs can be caused by the differences in the donors, tissues used, MSC subpopulations within a tissue, and in vitro culture conditions^21,28,29^. Indeed, there are studies showing that MSCs from adipose tissues are more stable and better than bone marrow MSCs in OA treatment^30,31^; that subpopulations of human MSCs expressing CD271 or CD146 are more effective on OA model mice^32,33^; and that MSCs without in vitro culturing show greater effectiveness than cultured MSCs, although conflicting results have been reported^34,35^. Nevertheless, the MSCs with great anti-OA activity await identification.

In this study, we compared the MSCs originated from the *Prrx1*- or *Dermo1*-lineage (all progeny cells of *Prrx1*-expressing cells or *Dermo1*-expressing cells during early development), which were isolated from the white adipose tissues (WAT) or dermal adipose tissues (referred to as dermis thereafter) of *Prrx1-Cre; R26*^*tdTomato*^ or *Dermo1-Cre; R26*^*tdTomato*^ mice. *Prrx1* and *Dermo1* have been used to mark early skeletal progenitor cells in the lateral plate of the mesoderm and *Prrx1* may mark earlier osteogenic progenitors than *Dermo1*^36–38^. We have recently shown that *Prrx1* is a genetic marker for adult skeletal stem cells and adipose stem cells^39^. We show here that *Prrx1* or *Dermo1* also marked various cell types in WAT and dermis, which gave rise to MSC cultures. We further show that the *Prrx1*-lineage MSCs from iWAT (inguinal WAT) are more potent in vitro than *Prrx1*-lineage MSCs from dermis or *Dermo1*-lineage MSCs from iWAT or dermis. Single cell profiling showed that iWAT *Prrx1*-lineage MSCs contained more adipose stem cell 2 (ASC2), the most primitive and potent adipose stem cells^39–41^, compared to *Dermo1*-lineage MSCs. We sorted *Prrx1*- or *Dermo1*-lineage MSCs from iWAT and dermis and tested their effectiveness on OA mouse models by articular cavity injection, without in vitro expansion. We found that only *Prrx1*-lineage MSCs from WAT showed significant therapeutic effect and they did so by differentiating into chondrocytes and incorporating into the damaged cartilage. They also inhibited inflammation and induced Col1α1 expression in resident chondrocytes, which represent fibrochondrocytes that may help articular cartilage regeneration^42–44^. Transcriptome analysis of the articular chondrocyte derived from the injected *Prrx1*-lineage MSCs showed that they had enriched expression in immune suppression, cell cycle, trophic factor, and chondrocyte related genes. This study has thus identified MSCs with great regenerative activity to treat OA.

## Results

### *Prrx1*- and *Dermo1*-lineages contribute to articular chondrocytes, WAT, and dermis

**Fig. 1:**
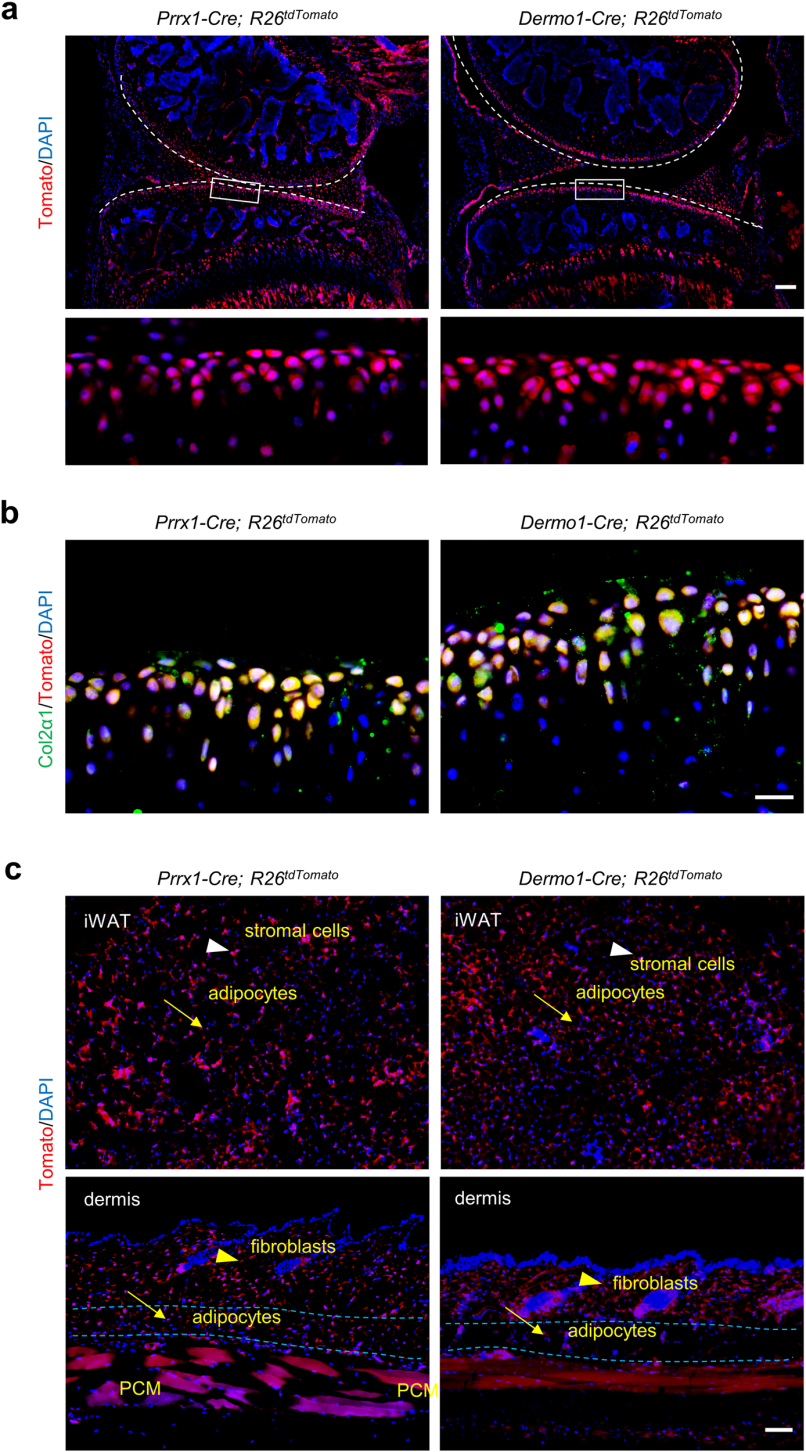
The *Prrx1*- and *Dermo1*-lineages contributed to articular cartilage, inguinal white adipose and dermal tissues. **a** Tracing of *Prrx1*- and *Dermo1*-lineage cells in normal articular cartilage in *Prrx1-Cre; R26*^*tdTomato*^ and *Dermo1-Cre; R26*^*tdTomato*^ mice. Scale bars = 200 μm. **b** Representative immunostaining results showed that *Prrx1-* and *Dermo1-*lineage cells in articular cartilage expressed Col2α1. Scale bars = 20 µm. **c** Tracing of *Prrx1-* and *Dermo1-*lineage cells in inguinal white adipose and dermis tissues. Scale bars = 100 μm. Upper panels: arrowheads, stromal cells; arrows: adipocytes, lower panels: arrowheads: dermal fibroblasts, arrows: adipocytes. PCM: panniculus carnosus muscle.

*Prrx1-Cre* and *Dermo1-Cre* mouse lines have been widely used to study skeletal development as they both label lateral plate of early mouse embryos^37,38^. We found that in adult *Prrx1-Cre; R26*^*tdTomato*^ and *Dermo1-Cre; R26*^*tdTomato*^ mice, chondrocytes at the articular cartilage of both the femur and tibia were Tomato^+^ (Fig. 1a), which expressed Col2α1, a marker for chondrocytes (Fig. 1b). In addition, osteoblasts in the femur and tibia were also Tomato^+^ (Fig. 1a). These results verified that the *Prrx1*-lineage or *Dermo1*-lineage give rise to articular cartilages and osteoblasts during development.

In adult *Prrx1-Cre; R26*^*tdTomato*^ and *Dermo1-Cre; R26*^*tdTomato*^ mice, we also observed large portions of Tomato^+^ cells in iWAT and dermis (Fig. 1c), suggesting that *Prrx1*-lineage and *Dermo1*-lineage contributed to these tissues. In iWAT, Tomato^+^ cells contained stromal cells and adipocytes and flow cytometry analysis revealed that they accounted for 8.19% and 9.87% of total cells in *Prrx1-Cre; R26*^*tdTomato*^ and *Dermo1-Cre; R26*^*tdTomato*^ mice, respectively (Supplementary Fig. 1a). In dermis, Tomato^+^ cells contained dermal fibroblasts and adipocytes and flow cytometry analysis revealed that they accounted for 7.98% and 11.51% of total cells in *Prrx1-Cre; R26*^*tdTomato*^ and *Dermo1-Cre; R26*^*tdTomato*^ mice, respectively (Supplementary Fig. 1a). These results suggest that both the *Prrx1* and *Dermo1* lineages contribute to WATs and dermis.

### *Prrx1*- and *Dermo1*-lineage MSCs have different differentiation potentials in vitro

**Fig. 2:**
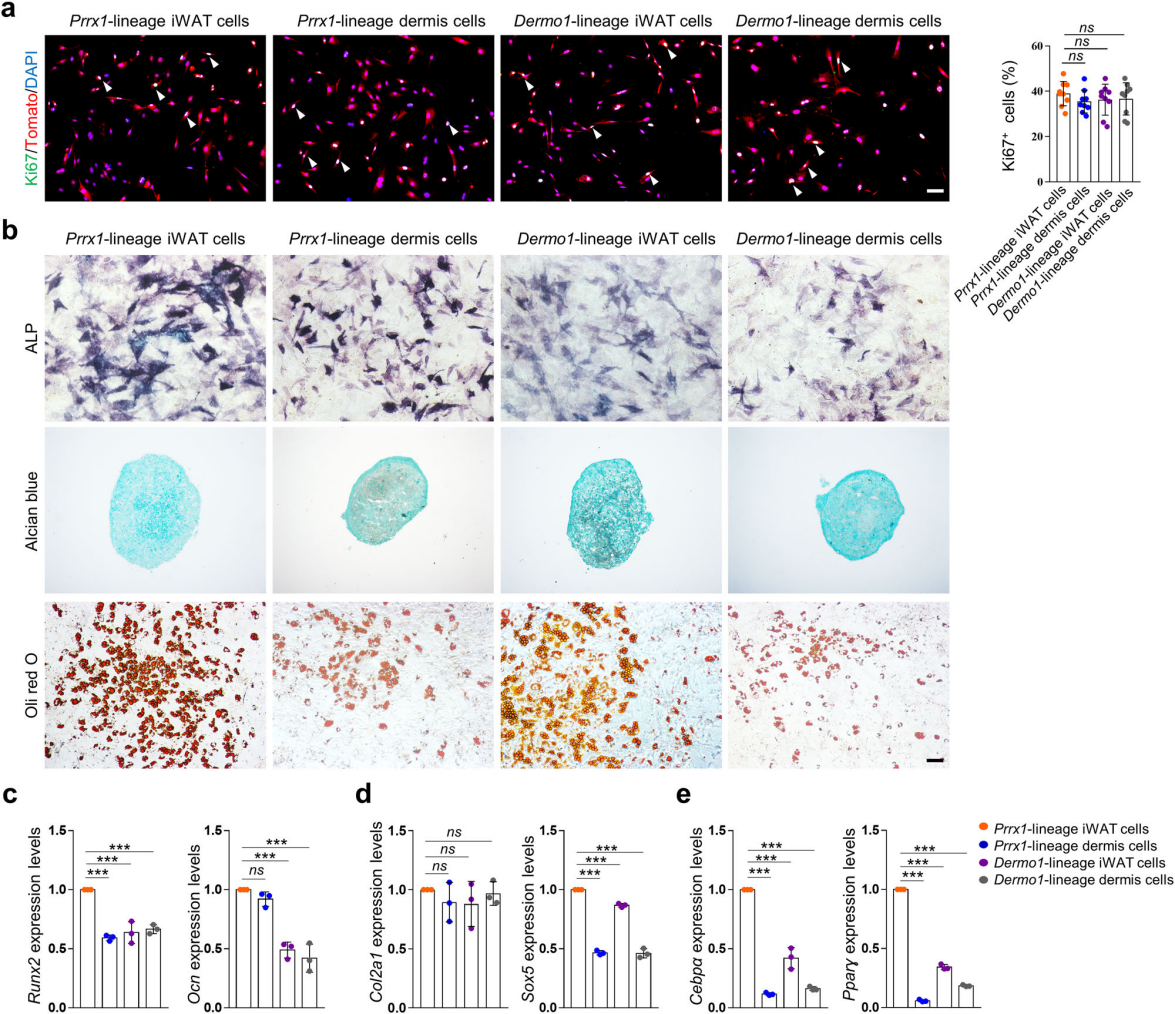
Comparison of proliferation and differentiation of *Prrx1*- and *Dermo1*-lineage cells in vitro. **a** Immunostaining of Ki67 in the four cell groups (cultured 5 days) in vitro. Right panel: Quantitation data of Ki67-positive cells. Scale bars = 100 µm. *N* = 3. Also see Supplementary Fig. 2 for separately-stained images. **b** Representative differentiation results for the four cell groups. Osteogenic differentiation was detected with ALP staining, chondrogenic differentiation was detected with Alcian blue staining, and adipogenic differentiation was detected with Oli red O staining. Comparison of the expression of cell type-specific marker genes. Quantitative PCR was carried out to determine the expression of osteoblast marker genes *Runx2* and *Ocn* (**c**), chondrocyte marker genes *Col2a1* and *Sox5* (**d**), and adipocyte marker genes *Cebpα* and *Pparγ* (**e**). The levels of marker genes in *Prrx1*-linege iWAT cells were set at 1.0. *N* = 3. Data are presented as means ± SEM in **a**, **c**–**e**. One-way ANOVA (and nonparametric) multiple comparisons was applied in **a**, **c**–**e**, *p* < 0.05 was considered as statistically significant. *p < 0.05, **p < 0.01, ***p < 0.001, and ****p < 0.0001. ns = not significant.

We isolated Tomato^+^ cells from iWAT or back skin dermis of *Prrx1-Cre; R26*^*tdTomato*^ mice or *Dermo1-Cre; R26*^*tdTomato*^ mice by FACS sorting. Smear examination of sorted cells confirmed that they were all Tomato^+^ (Supplementary Fig. 1b). Next, we cultured the four groups of Tomato^+^ cells and found that they adhered to the culture plates (Supplementary Fig. 2a) and underwent proliferation, manifested by Ki67 immunostaining results (Fig. 2a and Supplementary Fig. 2b). The in vitro proliferation rates of the four groups of cells were similar (Fig. 2a). We also carried out in vitro tri-lineage differentiation assays and found that *Prrx1*-lineage iWAT cells, *Prrx1*-lineage dermal cells, *Dermo1*-lineage iWAT cells, and *Dermo1*-lineage dermal cells could all differentiate into osteogenic, chondrogenic, and adipogenic cells (Fig. 2b). These results suggest that the Tomato^+^ cells from the *Prrx1* or *Dermo1* lineage have features of MSCs.

To quantify the differentiation potentials of these cells, we analyzed the expression of lineage specific markers. We used *Runx2* and *Ocn* (Osteocalcin) for osteoblasts, *Col2a1* and *Sox5* for chondrocytes, and *Cebpα* and *Pparγ* for adipocytes. Our quantitative PCR (qPCR) results showed that during osteogenic differentiation, *Runx2* expression was the greatest in *Prrx1*-lineage iWAT MSCs while *Ocn* expression in *Prrx1*-lineage iWAT MSCs and *Prrx1*-lineage dermal MSCs were superior to *Dermo1*-lineage iWAT MSCs and *Dermo1*-lineage dermal MSCs (Fig. 2c). In chondrogenic differentiation, while *Col2α1* expression was similar in the four MSC groups, *Sox5* expression was the highest in *Prrx1*-lineage iWAT MSCs, followed by *Dermo1*-lineage iWAT MSCs (Fig. 2d). In adipocyte differentiation, expression of both *Cebpa* and *Pparg* was the greatest in *Prrx1*-WAT MSCs, followed by *Dermo1*-iWAT MSCs (Fig. 2e). Overall, these findings indicate that *Prrx1*-lineage iWAT MSCs have the greatest stemness in vitro among the four groups.

We also compared the expression of cell surface markers in the four groups of cells. Flow cytometry analysis revealed that none of these cells expressed CD45, CD105, or CD106, yet they all expressed Sca-1, CD29, and CD44. The cell surface marker expression pattern was the same as most mouse MSCs, further supporting that *Prrx1*- and *Dermo1*- lineages give rise to WAT and dermal MSCs. Previous studies have shown that CD271^+^ or CD146^+^ human MSC subgroups have greater effectiveness on OA treatment^32,33^, we found that the mouse MSCs expressed non-detectable levels of CD271 and only a small portion of MSCs expressed CD146 (Supplementary Fig. 3a, b), suggesting that CD146 or CD271 cannot be used to separate the subpopulations of mouse MSCs. Overall, these results indicate that the four groups of MSCs have a similar cell surface marker expression pattern and future studies are needed to identify the cell surface markers that can distinguish these MSC subpopulations.

### scRNA-seq reveals that *Prrx1*-lineage cells contain more stem cells than *Dermo1*-lineage cells

**Fig. 3:**
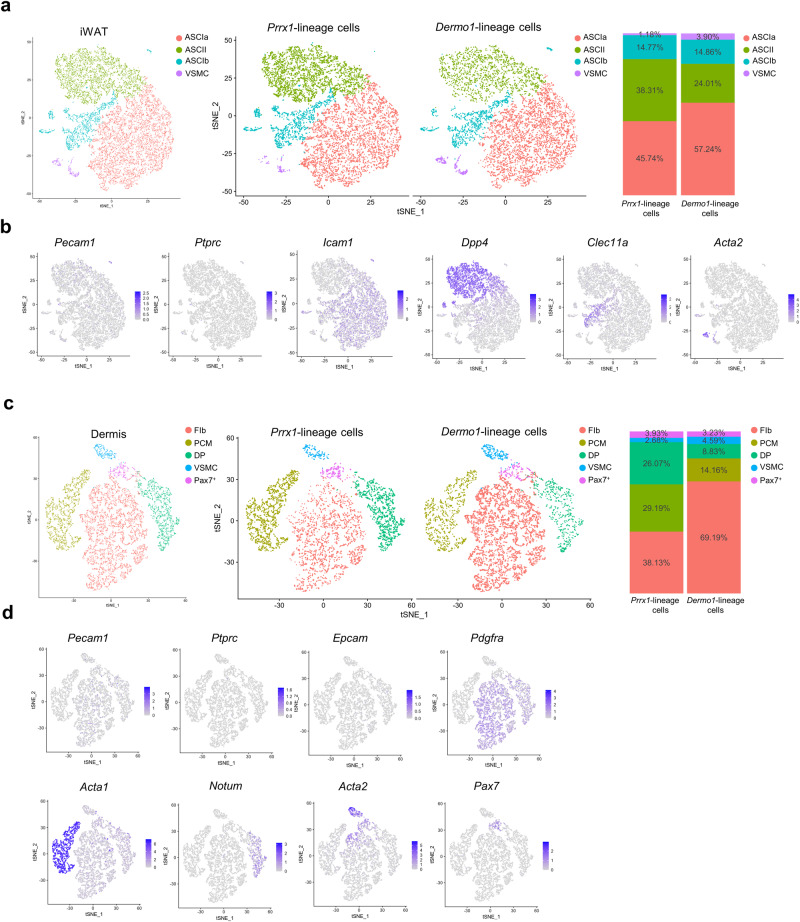
Single cell transcriptome analysis of the 4 MSC populations. **a** Unsupervised clustering of the iWAT Tomato^+^ cells isolated from *Prrx1-Cre; R26*^*tdTomato*^ and *Dermo1-Cre; R26*^*tdTomato*^ mice with tSNE. Right panel: the proportion of each cluster in iWAT. **b** The key marker gene expression in various iWAT cell clusters. **c** Unsupervised clustering of the dermis Tomato^+^ cells isolated from *Prrx1-Cre; R26*^*tdTomato*^ and *Dermo1-Cre; R26*^*tdTomato*^ mice with tSNE. Right panel: the proportion of each cluster in dermis. **d** The key marker gene expression in various dermis cell clusters.

We then analyzed *Prrx1*-lineage and *Dermo1*-lineage cells isolated from iWAT with single cell RNA-seq (scRNA-seq). Tomato^+^ cells from iWAT of 3 *Prrx1-Cre; R26*^*tdTomato*^ mice or *Dermo1-Cre; R26*^*tdTomato*^ mice were combined. Unbiased tSNE analysis showed that *Prrx1* or *Dermo1* marked adipose stem cell 2 (ASC2), ASC1a, ASC1b, and smooth muscle cells/pericytes, the major cell types in SVF and in MSCs derived from WAT (Fig. 3a, b). Moreover, *Prrx1* marked 59.6% more ASC2, the most primitive ASC population^40,41^, than the *Dermo1* counterpart, associated with a decrease in ASC1a, a adipose progenitor population. These results are consistent with the in vitro differentiation results and indicate that *Prrx1*-lineage MSCs contained more stem cells than the *Dermo1* counterpart.

We also performed scRNA-seq analyses of *Prrx1*-lineage or *Dermo1*-lineage cells isolated from the dermis. Tomato^+^ cells from dermis of 3 mice were combined. Unbiased tSNE analysis showed that *Prrx1* or *Dermo1* marked dermal fibroblasts, dermal papilla (DP) cells, panniculus carnosus muscle (PCM) cells including Pax7^+^ PCM, and vascular smooth muscle cells/pericytes (Fig. 3c, d). Moreover, *Prrx1* marked 195.4% more DP cells, which are known to have stem cell activities^45,46^, and fewer fibroblasts than the *Dermo1* counterpart. The difference in the cellular composition of WAT and dermis implies that MSCs derived from these two tissues may have different therapeutic effect on KOA (see later results).

### *Prrx1*-lineage WAT MSCs show the greatest therapeutic effect on OA models

**Fig. 4:**
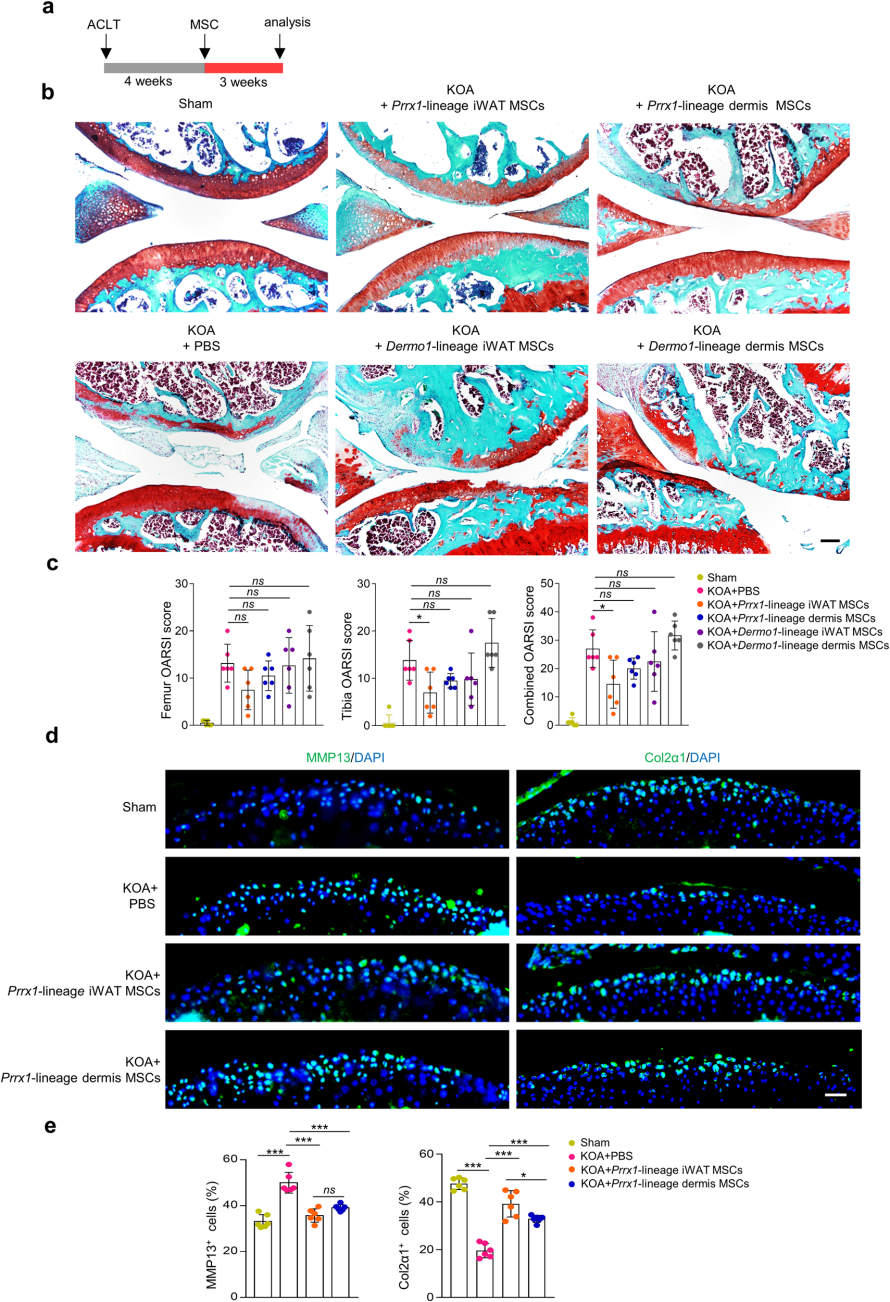
Comparison of the therapeutic effects of the 4 MSC groups on KOA. **a** Schematic diagram showing the experiment design. **b** Representative Safranin O staining results of the knee joint articular cartilage. Scale bars = 100 µm. **c** OARSI scores of the articular cartilage after treatment with MSCs. OARSI scores of femur (left), tibia (middle), and the combined (right). *N* = 6 mice. **d** Immunostaining for MMP13 and Col2α1 on the articular cartilage sections of mice. Scale bars = 50 µm. **e** Quantification results of MMP13 or Col2α1 positive cells. *N* = 6 mice. Data are presented as means ± SEM in **c** and **e**. One-way ANOVA (and nonparametric) multiple comparisons was applied in **c** and **e**, *p* < 0.05 was considered as statistically significant. *p < 0.05, **p < 0.01, ***p < 0.001, and ****p < 0.0001. ns = not significant.

We then used ACLT-induced OA model to test the efficacy of the four groups of MSCs. Based on previous studies^47,48^, we used 8-week-old *C57BL/6* male mice to induce OA on the right hind limbs, which showed destruction of articular cartilage associated with mild inflammation 4 weeks post the injury (Fig. 4a and later results). Next, we sorted the four groups of MSCs and directly injected them (one dose at 2.5 ×10^5^ cells) into the intra-articular cavity of the OA mice 4 weeks post ACLT (Fig. 4a), with the control group using PBS. Three weeks after MSC administration, the joints were analyzed and evaluated using the OARSI scoring system. The scores were calculated for the articular cartilage at the femur, tibia, or both. The effectiveness was ranked as *Prrx1*-lineage iWAT MSCs > *Prrx1*-lineage dermis MSCs = *Dermo1*-lineage iWAT MSCs > *Dermo1*-lineage dermis MSCs (Fig. 4b, c). Statistically, only the *Prrx1*-lineage iWAT MSCs showed significant effect on OA treatment (Fig. 4c).

### *Prrx1*-lineage iWAT MSCs slow down articular cartilage degradation

We also evaluated the therapeutic effects of MSCs by immunostaining of Matrix metalloproteinase 13 (MMP13) and Col2α1 on joint sections (Fig. 4d). In the following studies, we focused on *Prrx1*-lineage iWAT MSCs in comparison to the dermal counterpart due to their effectiveness on OA treatment. MMP13-mediated cartilage degradation is a critical step in OA pathogenesis. In the OA mice, we found that the number of cells expressing MMP13 was increased from 33.21% ± 1.18% to 50.00% ± 1.82% (*p* < 0.0001), whereas the number of cells expressing Col2α1 was reduced from 47.62% ± 0.98% to 19.59% ± 1.21% (*p* < 0.0001) (Fig. 4e). Treatment with *Prrx1*-lineage iWAT MSCs and *Prrx1*-lineage dermis MSCs reduced the numbers of cell expressing MMP13 from 50.00% ± 1.82% to 35.72% ± 1.19% (*p* < 0.0001) and 39.11% ± 0.56% (*p* = 0.0002), respectively, while the number of cell expressing Col2α1 was increased from 19.59% ± 1.21% to 39.19% ± 2.25% (*p* < 0.0001) and 32.91% ± 0.60% (*p* < 0.0001), respectively (Fig. 4e). These results suggest that *Prrx1*-lineage iWAT MSCs is better than *Prrx1*-lineage dermis MSCs in slowing down articular cartilage degradation, supporting the therapeutic effect of this MSC population.

We also tested whether *Prrx1*-lineage iWAT MSCs had a long-term therapeutic effect on KOA. We waited for 4 months instead of 3 weeks after MSC administration to the KOA mice. The therapeutic effect was still observed, although to a reduced extent (OARSI score from 46.3% to 26.6%) (Supplementary Fig. 4a–c). We also observed an anti-cartilage degradation effect by MSCs 4 months after administration (Supplementary Fig. 4d, e). These results suggest that these MSCs have a long-term therapeutic effect on OA. We then tested the effect of the *Prrx1*-lineage iWAT MSCs on KOA that was induced to develop for 8 weeks. Under this condition, even the *Prrx1*-lineage iWAT MSCs failed to show a significant effect (Supplementary Fig. 4f–h), suggesting that MSCs are effective only on early-stage OA but not late-stage OA.

### *Prrx1*-lineage MSCs differentiate into chondrocytes at the articular surface

**Fig. 5:**
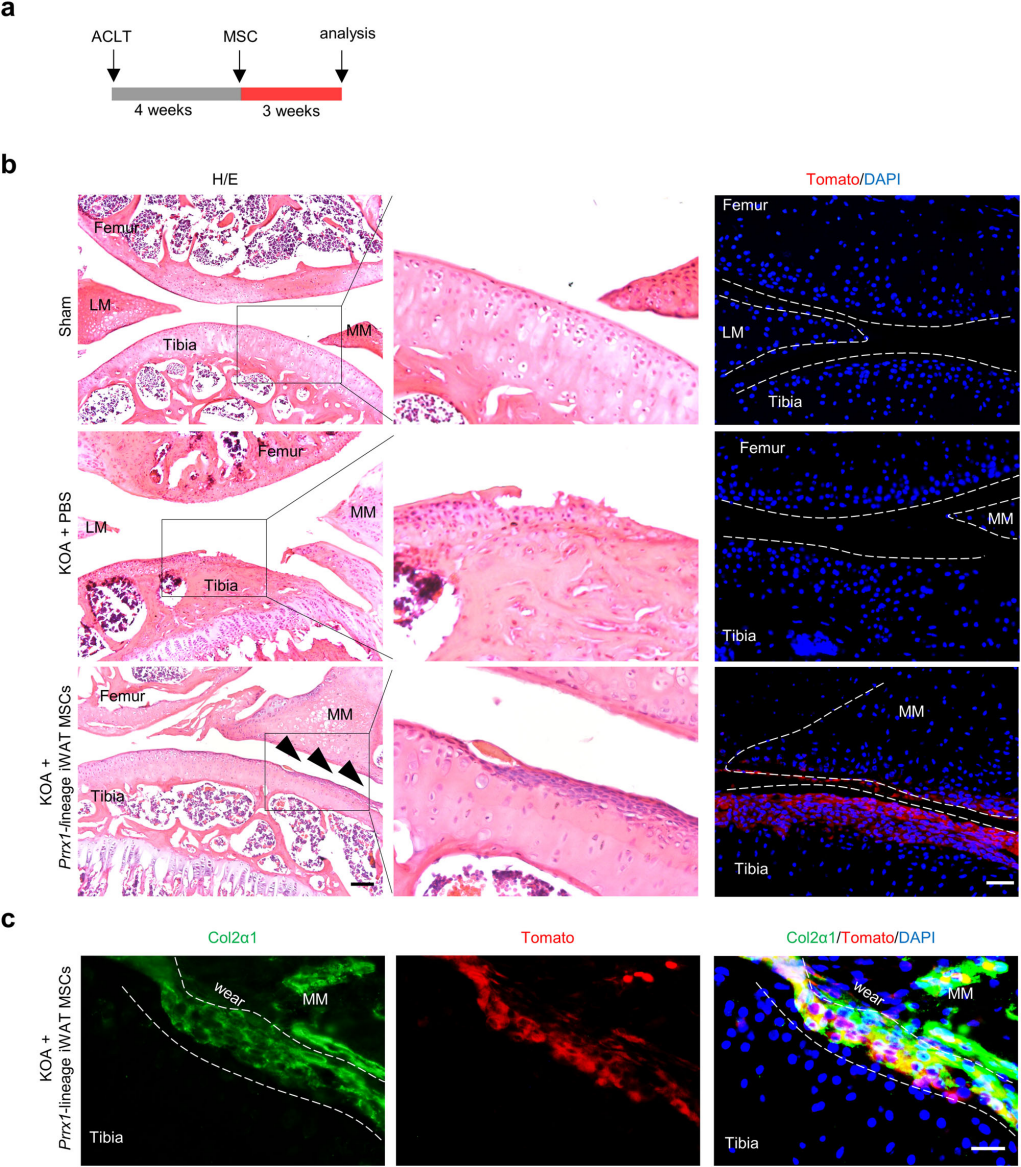
The injected *Prrx1*-linege iWAT MSCs differentiated into chondrocytes. **a** Schematic diagram showing the experiment design. **b** Tracing of *Prrx1*-lineage iWAT MSCs (Tomato^+^) three weeks after being injected into the intra-articular cavities. Sham, OA + PBS, and OA + *Prrx1*-linege iWAT MSCs groups were shown. Left panel: H/E staining, Scale bars = 100 µm. right panel: Tomato and DAPI staining, Scale bars = 50 µm. **c** Representative immunostaining results for Col2α1 in articular cartilage. Scale bars = 20 µm. MM medial meniscus, LM lateral meniscus.

We also traced the injected *Prrx1*-lineage iWAT MSCs, which were Tomato^+^, in the joints of OA mice (Fig. 5a). We detected Tomato^+^ cells on the surface of articular cartilage (Fig. 5b). As seen in KOA joint sections, the incorporated Tomato^+^ cells accounted for approximately 25% of femur or tibia articular surface, indicating that the injected MSCs incorporated into the damage cartilage. We immunostained the sections with antibodies against Col2α1 and found that the Tomato^+^ cells on the articular surface were largely positive for Col2α1 (Fig. 5c). On the other hand, *Prrx1* lineage cells derived from the dermis, which are only secondary to *Prrx1* lineage cells from iWAT in the effectiveness on KOA treatment, did not show incorporation into the injured articular cartilage (Supplementary Fig. 5). These results suggest that the *Prrx1*-lineage iWAT MSCs differentiated into chondrocytes at the surface of articular cartilage, this would help to repair the damaged cartilage.

### *Prrx1*-lineage iWAT MSCs also act on resident chondrocytes

In addition, we observed expression of Col1α1 in the MSC-treated articular cartilage. However, the Col1α1 signals did not co-localize with Tomato^+^ cells, instead, expression of Col1α1 occurred in the resident articular chondrocytes (Supplementary Fig. 6a). Quantitative PCR confirmed an increase in *Col1a1* mRNA levels in the articular cartilage samples with MSC treatment (Supplementary Fig. 6b). Interestingly, even in the articular surface without incorporation of *Prrx1*-lineage MSCs, we observed high level of expression of Col1α1 (Supplementary Fig. 6a). Overall, expression of Col1α1 was observed at nearly 50% of the injured cartilage in the *Prrx1*-lineage iWAT MSCs-treated mice. On the other hand, articular cartilage in normal mice contained no Col1α1^+^ chondrocytes (Supplementary Fig. 6c). Although articular cartilage contained some Col1α1^+^ chondrocytes in KOA mice, Col1α1 expression was largely lost due to the abrasion of the articular cartilage under our experimental settings (Supplementary Fig. 6d). Thus, administrated MSCs greatly promoted Col1α1 expression in resident chondrocytes in OA mice, which may be induced by trophic factors secreted by injected MSCs. Single cell profiling have identified Col1α1^+^ fibrochondrocytes in articular cartilage besides the subchondral osteoblasts and the meniscus in the knee joint^49,50^. Col1α1 expression in chondrocyte is reported to be an indication of dedifferentiation during regeneration^51,52^, and may help MSC migration and adhesion and promote cartilage repair^44,53,54^. Certainly, this warrants further investigation.

### *Prrx1*-lineage MSCs also suppressed inflammation

**Fig. 6:**
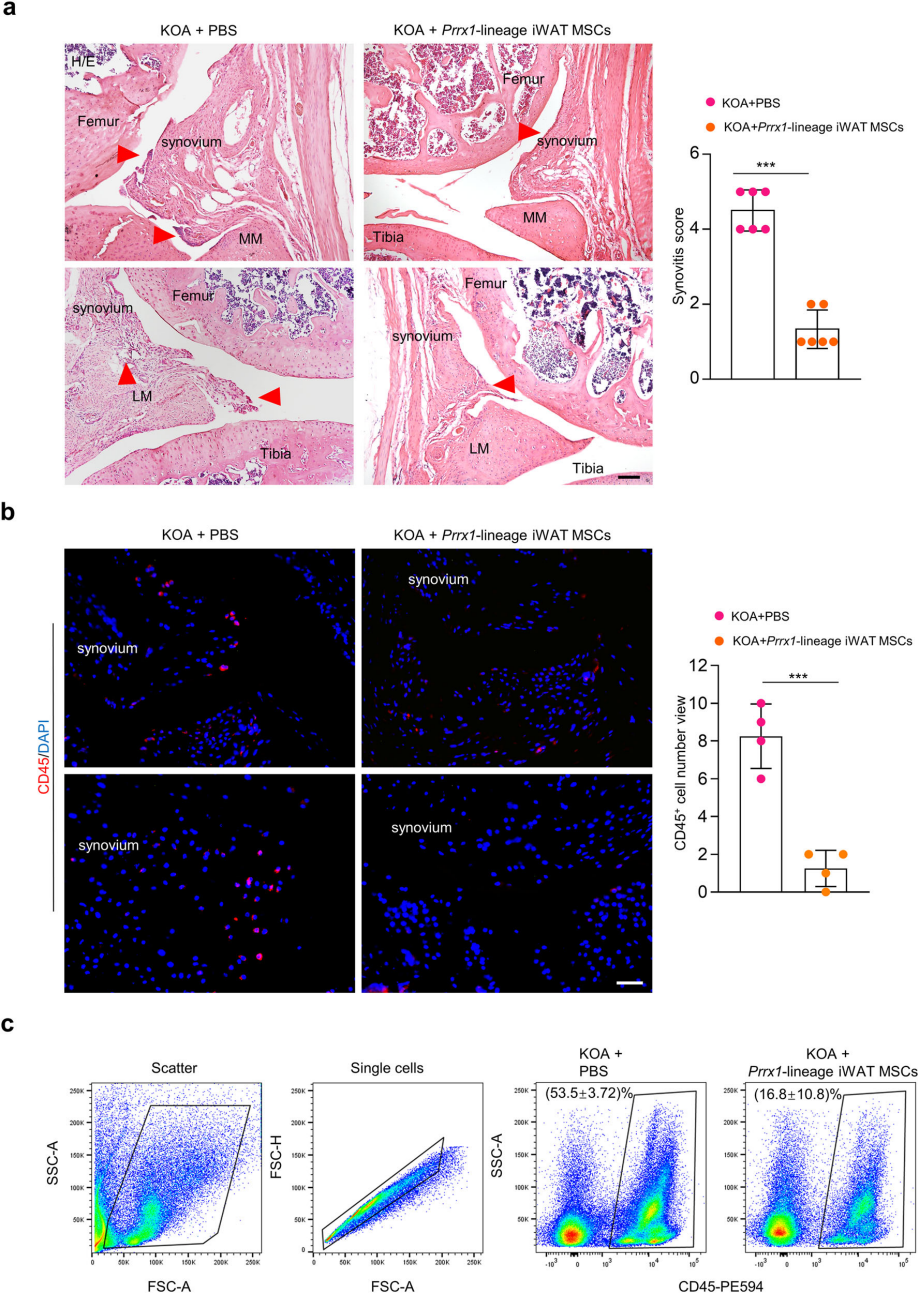
*Prrx1*-linege MSCs inhibited synovial inflammation in KOA mice. **a** H/E staining of the knee joint sections of the OA mice and OA mice treated with *Prrx1*-linege iWAT MSCs. Arrowhead: inflammation. MM medial meniscus, LM lateral meniscus. Scale bars = 100 µm. Right panel: Quantitation data for synovitis score. *N* = 6 mice. **b** Immunostaining for CD45 on the joint sections of mice. Scale bars = 50 µm. Right panel: Quantitation data for CD45^+^ cells. *N* = 4 mice. **c** Flow cytometry analysis of the proportion of CD45^+^ cells in synovium. *N* = 3 mice. Data are presented as means ± SEM. Unpaired two-tailed Student’s *t* test were applied, *p* < 0.05 was considered as statistically significant. *p < 0.05, **p < 0.01, ***p < 0.001, and ****p < 0.0001.

In addition, we detected mild inflammation at the synovium in the knee joint of OA model mice (Fig. 6a), which is an established pathologic factor^55^. We found that *Prrx1*-lineage iWAT MSCs could diminish inflammation in the synovium (Fig. 6a). We also performed immunostaining for CD45^+^ immune cells and found that *Prrx1*-lineage iWAT MSCs resulted in a reduction of CD45^+^ cells in the synovium (Fig. 6b). Flow cytometry analysis confirmed this observation (Fig. 6c). These results, taken together, suggest that the *Prrx1*-lineage MSCs also dampen inflammation in the OA joints.

### Transcriptome analysis of the articular chondrocytes derived from *Prrx1*-lineage MSCs

**Fig. 7:**
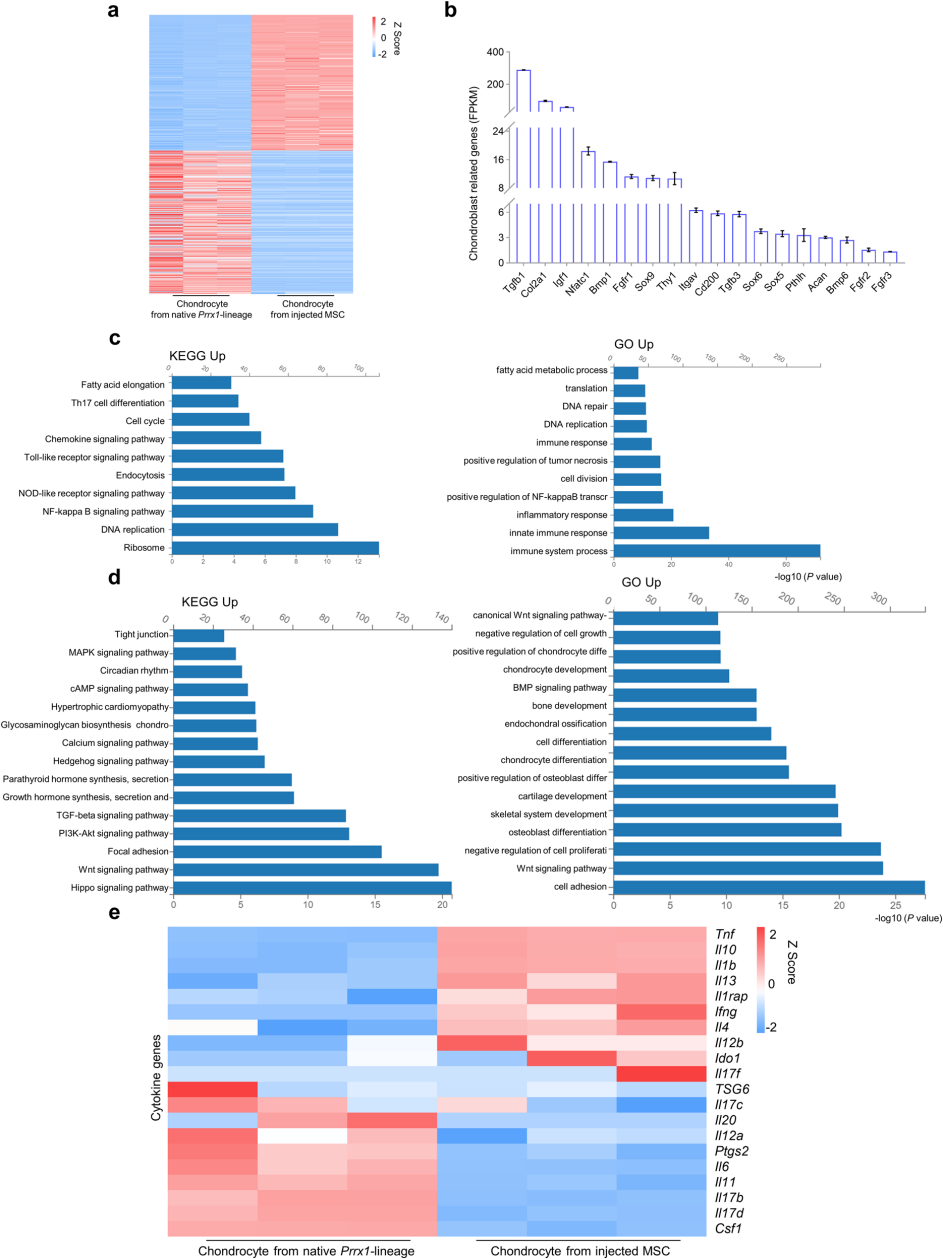
Analysis of the transcription profiles of articular chondrocytes derived from *Prrx1*-linege iWAT MSCs. **a** Heatmaps of the top 500 genes expressed in articular chondrocytes derived from *Prrx1*-linege iWAT MSCs and endogenous articular chondrocytes of *Prrx1-Cre; R26*^*tdTomato*^ mice. *N* = 3 mice. **b** Chondroblast related genes (FPKM) expressed by articular chondrocytes derived from *Prrx1*-lineage iWAT MSCs. **c** KEGG (up-regulated) and GO (up-regulated) analysis results of the articular chondrocytes derived from *Prrx1*-linege iWAT MSCs. **d** KEGG (up-regulated) and GO (up-regulated) analysis results of the endogenous articular chondrocytes in *Prrx1-Cre; R26*^*tdTomato*^ mice. **e** Heatmaps of the cytokine genes expressed in articular chondrocytes derived from *Prrx1*-linege iWAT MSCs and endogenous articular chondrocytes of *Prrx1-Cre; R26*^*tdTomato*^ mice. *N* = 3 mice.

We then isolated Tomato^+^ cells from the repaired articular cartilage of the KOA mice (*n* = 3), which were derived from injected MSCs, and performed bulk RNA-seq using endogenous articular Tomato^+^ chondrocytes of *Prrx1-Cre; R26*^*tdTomato*^ mice as a control. We observed a difference in the transcriptomes of the two cell populations (Fig. 7a). The articular Tomato^+^ articular chondrocytes derived from the injected MSCs showed expression of various chondrocyte-specific genes (Fig. 7b), as well as enriched expression of genes that regulate cell cycle, DNA replication/repair, inflammation, and lipid metabolism (Fig. 7c and Supplementary Fig. 7a). On the other hand, the endogenous Tomato^+^ cells showed enriched expression in Hedgehog signaling, TGFβ signaling, Wnt signaling, BMP signaling, chondrocyte differentiation, and skeletal development (Fig. 7d). Further analysis showed that Tomato^+^ articular chondrocytes derived from the injected MSCs expressed some cytokines that had anti-inflammatory activities, including IL1ra, IL4, IL10, IL13, and TGFβ besides IDO, TSG6, and PGE2 (Ptgs2) (Fig. 7e). In addition, articular Tomato^+^ cells derived from the injected MSCs expressed some trophic factors including IGF1 and VEGF in addition to previously reported HGF, TGFβ2, and FGFs (Supplementary Fig. 7b). These results support that the injected MSCs could differentiate into articular chondrocytes and express anti-inflammatory factors and trophic factors.

## Discussion

OA is an incurable common disease and the emerging MSC-based therapy has shown some promise in OA treatment^56^. However, in spite of more than 200 clinical trials, predictable, reliable, and efficacious MSC products remain in shortage^57^. The problems mainly arise from the heterogeneity of MSCs and the uncertain mechanisms of action^58^. This study compared the therapeutic effects of *Prrx1*-lineage and *Dermo1*-lineage MSCs isolated from white and dermal adipose tissues on OA mouse models. The isolated *Prrx1*-lineage and *Dermo1*-lineage MSCs were directly injected into the articular cavity of OA model mice, without in vitro expansion. We found that the *Prrx1*-lineage iWAT cells are superior to other three groups of MSCs in OA treatment and show long-term effect. *Prrx1*-lineage iWAT MSCs suppress the expression of MMP13 and promote the expression of Col2α1 in OA cartilage samples, suggesting that cartilage destruction is suppressed.

MSCs from various anatomic sources are used in preclinical and clinical trials to test their effectiveness against OA, including MSCs from the bone marrow, WAT, and umbilical cord. In our study, we find that MSCs from the white adipose tissue are better than MSCs from dermal adipose tissue. This difference is likely caused by the fact that MSCs isolated from dermis mainly contain dermal fibroblasts, as evidenced by our scRNA-seq data, which may be less potent than MSCs of adipose tissues. In human, the dermal fat and the subcutaneous fat at the abdomen are continuous and are difficult to separate. Based on our findings, MSCs from the subcutaneous adipose tissues (the deep layer) may be better than those from the dermis.

MSCs from the same tissue can be heterogeneous as suggested by single cell profiling and genetic tracing studies^42,59^. Both *Prrx1-Cre* and *Dermo1-Cre* mouse lines have been used to study skeletal development. However, previous studies have reported that *Prrx1*-marked cells showed properties not shared by *Dermo1*-marked cells. For example, ablation of *Patched1*, which activates the Hedgehog signaling, in *Prrx1*-lineage cells leads to development of osteosarcoma and chondroma whereas ablation of *Patched1* in *Dermo1*-lineage cells fails to do so^60^. In addition, ablation of *Tsc1*, which leads to activation of the mTOR signaling, in *Prrx1*-lineage cells leads to greatly increased bone mass while ablation of *Tsc1* in *Dermo1*-lineage cell leads to mild increase in bone mass^61^. Here, we show that both *Prrx1* and *Dermo1* mark white adipose and dermal adipose tissues, and give rise to adherent cells that have properties of MSCs. Notably, *Prrx1*-lineage iWAT MSCs are more potent than *Dermo1*-lineage iWAT MSCs. Our single cell profiling revealed that *Prrx1*-lineage MSCs contained more stem cells (ASC2) and fewer adipose progenitors than *Dermo1*-lineage MSCs. These findings suggest that ASC2 cells, which have the greatest stemness among the SVF cells, may be a better candidate for OA treatment. Future studies may isolate the ASC2 cells and test their effect on OA mice. Moreover, our findings suggest that the in vitro and in vivo stemness of MSCs is an indicator for their effectiveness in OA treatment, at least for MSCs with regenerative abilities.

We show that *Prrx1*-lineage MSCs might exert their therapeutic effects in multiple ways. While it is generally believed that the function of MSCs are mainly mediated by paracrine secretion of immunomodulatory cytokines and trophic factors, we find that *Prrx1*-lineage iWAT MSCs may treat OA with 3 different mechanisms: differentiating into Col2α1^+^ chondrocytes and incorporating into the articular cartilage; inducing Col1α1 expression in resident chondrocytes, and suppressing synovial inflammation. Transcriptome analyses confirmed these features in the articular chondrocytes derived from the injected MSCs. Single cell profiling have identified Col1α1^+^ fibrochondrocytes in articular cartilage of OA samples besides subchondral osteoblasts and meniscus in the knee joint^49,50^. The Col1α1^+^ cells in meniscus have stem cell activities^42,50^, while Col1α1^+^ fibrochondrocytes in cartilage may help repair cartilage^44^, although this needs to be verified^62,63^. Col1α1 expression may be stimulated by MSCs-secreted trophic factors including IGF1 and VEGF in addition to previous reported HGF, TGFβ2, and FGFs^21^. In addition, the *Prrx1*-lineage WAT MSCs may suppress synovial inflammation with new mechanisms. We find that these cells synthesize IL1ra, IL4, IL10, IL13, and TGFβ to suppress inflammation besides IDO, TSG6, and PGE2.

Our study identified a genetically-marked MSC population that can regenerate the damaged articular cartilage, especially in early stages of OA, in addition to suppressing inflammation and secreting trophic factors. The regenerating ability of MSCs appears to rely on the stemness of the cells and last for at least 4 months.

## Methods

### Study design

The aim of this study is to compare the therapeutic effects of *Prrx1* and *Dermo1* lineage MSCs from white adipose tissues and dermal adipose tissues on knee Osteoarthritis (KOA) mouse models to identify MSC subpopulations with the greatest therapeutic effect in OA treatment. We isolated *Prrx1*- and *Dermo1*-lineage MSCs by FACS sorting (Tomato^+^) from iWAT and dermis of *Prrx1-Cre; R26*^*tdTomato*^ and *Dermo1-Cre; R26*^*tdTomato*^ mice and compared their in vitro proliferation, tri-lineage differentiation potentials, the expression of cell surface markers, and their scRNA-seq signatures, to determine their stemness. The mice were divided to six groups randomly (Sham, OA + PBS, OA + *Prrx1*-lineage iWAT MSCs, OA + *Prrx1*-lineage dermal MSCs, OA + *Dermo1*-lineage iWAT MSCs, and OA + *Dermo1*-lineage iWAT MSCs, each group had six mice). We directly injected one dose of MSCs (2.5 × 10^5^ cells in 25 μl PBS) into the articular cavity of OA model mice, which are induced by ACLT. The mice used in each experiment was indicated in the figure legends. Sample size was determined on the basis of previous experience.

### Mice maintenance

The *Prrx1-Cre*, *Dermo1-Cre*, and *R26*^*tdTomato*^ mouse lines were purchased from the Jackson Laboratory (https://www.jax.org/cn/). The *Prrx1-Cre; R26*^*tdTomato*^ and *Dermo1-Cre; R26*^*tdTomato*^ mouse lines are on *C57BL/6* background. The normal mice used to create knee arthritis models are also on *C57BL/6* background and were all purchased from The Nanjing model animal center. The mice used in this study were all male as most previous studies used male mice^47,48^. The mice used in the experiment were anesthetized using 40 mg kg^−1^ sodium pentobarbital by intraperitoneal injection, to obtain tissue sample or euthanized by carbon dioxide inhalation.

Animal experiments were carried out in accordance with recommendations in the National Research Council Guide for Care and Use of Laboratory Animals and in comply with relevant ethical regulations for animal testing and research, with the protocols approved by the Institutional Animal Care and Use Committee of Shanghai, China (SYXK(SH)2011-0112). This study follows the project that “Studies on the mechanism of MSC self-renewal differentiation and regulation of related tissue stem cells”, which was approved by Shanghai Jiao Tong University in 2015 and the approval number is A2015027. In this study, only corresponding authors and Hongshang Chu were aware of the group allocation of the experiments (during the allocation, the conduct of the experiment, the outcome assessment, and the data analysis).

### Cell flow cytometry analysis and cell sorting

The dermis, iWAT, synovial tissue, and knee cartilage were taken from the mice. The dermis was digested with elastase and type IV collagenase, while inguinal white adipose tissue was digested with type II collagenase, synovial tissue was digested with type II collagenase. The knee cartilage was digested with type II collagenase. The released cells were passed through 40 μl and 70 μl cell sieves to get single cells. Then, we sorted out Tomato^+^ cells using Bio-Rad Flow Cytometer.

For cell surface marker expression with flow cytometry, the following antibodies are used: Sca-1-FITC (Biolegend, Cat #: 108105, Clone: D7, 1:50 dilution), CD29-FITC (Biolegend, Cat #: 102205, Clone: HMβ1-1, 1:50 dilution), CD44-APC (Biolegend, Cat #: 103011, Clone: IM7, 1:200 dilution), CD45-FITC (Biolegend, Cat #: 103107, Clone: 30-F11, 1:200 dilution), CD45-PE594 (Biolegend, Cat #: 103145, Clone: 30-F11, 1:200 dilution), CD73-APC (Biolegend, Cat #: 127209, Clone: TY/11.8, 1:200 dilution), CD146-APC (Biolegend, Cat #: 134711, Clone: ME-9F1, 1:200 dilution), CD105-AF488 (Biolegend, Cat #: 120405, Clone: MJ7/18, 1:250 dilution), CD106-FITC (Biolegend, Cat #: 105705, Clone: 429(MVCAM.A), 1:200 dilution), and CD271-FITC (Biolegend, Cat #: 345103, Clone: ME20.4, 1:100 dilution).

### In vitro MSC proliferation and differentiation

The Tomato^+^ cells were cultured in vitro to determine the proliferation ability by quantitating the expression of Ki67 with an immunofluorescence staining kit (Abcam, ab15580) and DAPI (ThermoFisher Scientific). The cell cultures were also used to determine the differentiation ability. They were induced to differentiate into osteogenic, chondrogenic, and adipogenic cells. For osteogenic differentiation, the MSCs were seeded at 5 × 10^3^/well in 12-well plates. The next day, the cells were switched into osteogenic medium (α-MEM medium containing 15% FBS, 10 mM β-glycerol phosphate, and 50 μg/ml ascorbic acid) for 7–10 days, with medium changed every 2 days. The cells were then fixed in 4% paraformaldehyde and stained with ALP. For chondrogenic differentiation, the cells were suspended at a concentration of 1.0 × 10^7^ cells/ml. We transferred the cells into 1.5 ml RNA-free EP tubes for centrifugation using 2.6 g centrifugal force, gathered the cells at the bottom of EP tubes, and discarded the supernatant. After adding α-MEM containing 15% FBS, 100 IU/ml penicillin, and 100 μg/ml streptomycin, the EP tube was placed in a cell incubator in a semi-closed state for culture for 48 h, and after cell agglomeration, the medium was discarded and chondrogenic medium (α-MEM containing 15% FBS, 100 nM dexamethasone, 10 ng/ml TGFβ1, and 1 μM ascorbate-2-phosphate) was added. The cell mass was maintained for 21 days, with the medium changed every 3 days and the cell mass was fixed with paraformaldehyde and sliced, which were lastly stained with Alcian blue. For adipocyte differentiation, the MSCs were seeded at 2 × 10^4^/well in a 12-well plate and cultured in α-MEM containing 15% FBS, 100 nM dexamethasone, and 5 μM insulin for 2 days. Then, the cells were switched into adipocyte maintenance medium (α-MEM containing 15% FBS and 5 μM insulin) for 6 days, with medium changed every3 days. The cells were then fixed and stained with Oil red O solution.

### RNA isolation and quantitative PCR

Differentiation and expression were also assessed with quantitative PCR. Total RNA was extracted from the articular cartilage or cells, which had been induced to differentiate into osteogenic, chondrogenic, and adipogenic, using Trizol reagent (Invitrogen) and was reverse transcribed using PrimerScriptTM RT reagent Kit (TaKaRa, RR037A) to obtain cDNA. Quantitative PCR was performed using the Roche Light Cycler 480II Assay system (Roche). The levels of different mRNA species were calculated with the delta‐delta CT method and normalized to GAPDH. The primer sequences are used in Supplementary Table 1.

### Surgically-induced osteoarthritis mouse model and MSC injection

All animals were treated according to standard guidelines approved by the Shanghai Jiao Tong University ethics committee. OA was induced by ACLT in 8-week-old *C57BL/6* male mice, which gradually developed KOA pathology starting from 4 weeks, as previous reported^47,48^. Each mouse is treated as the experimental unit. The mice were randomly divided to six groups (Sham, OA + PBS, OA + *Prrx1*-lineage iWAT MSCs, OA + *Prrx1*-lineage dermis MSCs, OA + *Dermo1*-lineage iWAT MSCs, and OA + *Dermo1*-lineage dermis MSCs). The mice were all maintained in the same room and same access to water and food. They were derived from the same breeder to minimize potential confounders. Intra-articular injections of 1 × PBS or MSCs (2.5 × 10^5^ cells in 25 μl PBS) were carried out at 4 or 8 weeks post-KOA surgery with German Braun Disposable Sterile Insulin Syringe (0.3*8 mm, 1 ml).

### Histological analysis, immunohistochemistry, and immunofluorescence staining

The harvested knee joints at 3 weeks or 4 months post-surgery, which were fixed in 4% (vol/vol) neutral buffered formalin for 24 h and decalcified in neutral 10% (wt/vol) EDTA solution for 1 month at room temperature on the oscillator. The samples were then dehydrated, cleared, and embedded in paraffin blocks sequentially, or soaked in 20% sucrose and then 30% sucrose and finally embed with optimal cutting temperature compound (OCT) in liquid nitrogen. The paraffin-embedded tissues were cut 10 μm with a paraffin microtome and the frozen-embedded tissues were cut 8 μm with a cryostat. Paraffin sections were used for safranin-O and H/E staining while frozen sections were used for immunostaining. Mice samples (*n* = 6) from each group were evaluated by the Osteoarthritis Research Society international (OARSI) scoring system with a score of 0 standing for normal cartilage, 0.5 = loss of proteoglycan with an intact surface, 1 = superficial fibrillation without loss of cartilage, 2 = vertical clefts and loss of surface lamina (any % or joint surface area), 3 = vertical clefts/erosion to the calcified layer lesion for 1%–25% of the quadrant width, 4 = lesion reaches the calcified cartilage for 25%–50% of the quadrant width, 5 = lesion reaches the calcified cartilage for 50%–75% of the quadrant width, and 6 = lesion reaches the calcified cartilage for 75% of the quadrant width. The synovitis score was based on the method in published articles, all defined histopathological qualities are graded from absent (0), slight (1) and moderate (2) to strong (3), with summaries ranging from 0 to 9. 0 to 1 corresponds to no synovitis (inflammatory grade = 0), 2 to 3 to a slight synovitis (inflammatory grade 1), 4 to 6 to a moderate synovitis (inflammatory grade 2), and 7 to 9 to a strong synovitis (inflammatory grade 3)^64^. The cartilage sections were incubated overnight with polyclonal anti-Col2α1 antibody (abcam, Cat #: ab34712, rabbit, 1:50 dilution), anti-MMP13 antibody (abcam, Cat #: ab39012, rabbit, 1:100 dilution), anti-Col1α1 antibody (abcam, Cat #: ab21286, rabbit, 1:100 dilution), anti-Aggrecan antibody (millipore, Cat #: AB1031, rabbit, 1:50 dilution), anti-Col1α1 antibody (proteintech, Cat #: 67288-1, mouse, 1:100 dilution), and anti-CD45 antibody (abcam, Cat #: ab10558, rabbit, 1:100 dilution). The sections were then incubated with secondary antibodies conjugated with Alexa Fluor IgG(H + L) 488 (invitrogen, Cat #: A11008, goat anti-rabbit, 1:100 dilution) or Alexa Fluor IgG(H + L) 555 (invitrogen, Cat #: A11001, goat anti-mouse, 1:100 dilution). Slides were mounted with anti-fade mounting medium (OriGene, Cat #: Zli-9556, 1:1 dilution) and DAPI (ThermoFisher Scientific, Cat #: 62248, 1:1000 dilution). Images were taken under Olympus DP72 microscope (Olympus Microsystems).

### scRNA-seq and analysis

Isolated Tomato^+^ cells from iWAT and dermis were used for single cell RNA sequencing by10X Genomics. RNA from the barcoded cells was subsequently reverse-transcribed and sequencing libraries constructed with reagents from a Chromium Single Cell 3’ v3 reagent kit (10X Genomics) following the manufacturer’s instructions. Sequencing was performed with Illumina NovaSeq 6000 (Illumina). Raw reads were demultiplexed and mapped to the mouse reference genome by Cell Ranger version 6.0.2 (10X Genomics) pipeline using default parameters. Each tissue were sequenced at a depth of about 70% saturation. The generated gene-cell expression matrices were used for subsequent analysis in R version 4.3.1 using Seurat version 4.3.0.1. “Cells” fit any of the following criteria were excluded: <200 expressed genes, >20% UMIs mapped to mitochondria. Samples from iWAT or dermis were respectively integrated using “FindIntegrationAnchors” and “IntegrateData” functions. Integrated data were undergone standard cell cycle regression process provided by Seurat. Processed data were used for downstream graph-based clustering and t-SNE visualization. “FeaturePlot” function in Seurat was used for the visualization of Specific genes expression.

### Bulk RNA-seq and analysis

We harvested Tomato^*+*^ articular chondrocytes formed by injected *Prrx1*-lineage iWAT MSCs from KOA mice and age -and sex-matched *Prrx1-Cre; R26*^*tdTomato*^ mice. Three mice were used in either sample. RNA-seq was performed by BGI using the Illumina system. Bulk RNA-Seq data analysis was performed as follows: (1) Data filtering, the raw data obtained from sequencing was filtered using SOAPnuke (v1.5.6) to filter out 1) reads containing adapter (adapter contamination); 2) reads with an unknown base N content greater than 5%, and 3) low-quality reads (reads with a mass value of less than 15 and more than 20% of the total base number of the reads are low-quality reads) to obtain clean data. Follow-up use of Dr. Tom’s Multi-Omics Data Mining (https://biosys.bgi.com) Department conducts data analysis, mapping and mining. (2) Differential gene analysis was performed, and the clean data was aligned to the reference genome using HISAT2 (v2.1.0) software. Use Bowtie2 (v2.3.4.3) to align the clean data to the reference gene set. Gene expression quantification was performed using RSEM (v1.3.1) software, and clustering heat maps of gene expression in different samples were plotted using pheatmap (v1.0.8). Differential gene testing was performed using DESeq2 (v1.4.5) with Q value ≤ 0.05 or FDR ≤ 0.001. (3) KEGG and GO enrichment analysis, Phyper was used to perform GO (http://www.geneontology.org/) and KEGG (https://www.kegg.jp/) enrichment analysis of differential genes, with Qvalue ≤ 0.05 as the threshold, and the definition of meeting this condition was significant enrichment in candidate genes.

### Statistical analysis

All the measurements were collected by two authors who do not know the allocations of mice. The FlowJo software was used to analyze and plot the proportion of flow sorted cells. The GraphPad Prism 8.0.2 software was used to analyze and plot the qPCR and immunofluorescence results. All quantitative data are presented as means ± SD unless indicated otherwise. One-way ANOVA (and nonparametric) multiple comparisons and Unpaired two-tailed Student’s *t* test were applied to evaluate the correlation data and *p* < 0.05 was considered as statistically significant. *p < 0.05, **p < 0.01, ***p < 0.001, and ****p < 0.0001. We ensure that we have not set criteria and exclusions in this study. For each analysis, we have reported the exact value of *n* in each experimental group.

### Supplementary information

Supplementary table and figures

nr-reporting-summary

## Data Availability

All data generated in this study are included in this published article or the supplementary information files. The bulk RNA-seq data have been deposited into NCBI (GSE253195). The scRNA-seq data have been deposited into NCBI (PRJNA1063441). The materials and detailed protocols are available by contacting Baojie Li at libj@sjtu.edu.cn.

## Code availability

The datasets in this work were generated or processed with publicly available software. All the details of the analysis codes used in the manuscript are provided in methods.
