# Supplementary material for: Comparison studies identify mesenchymal stromal cells with potent regenerative activity in osteoarthritis treatment
Source: NPJ Regen Med. 2024 Apr 1;9:14. doi: 10.1038/s41536-024-00358-y (PMC10984924; doi:10.1038/s41536-024-00358-y)
Supplement: Supplementary file 1 — Supplementary table and figures [file 41536_2024_358_MOESM1_ESM.pdf]

Supplementary information

Comparison studies identify mesenchymal stromal cells with potent regenerative activity in osteoarthritis treatment

Hongshang Chu[1], Shaoyang Zhang[1], Zhenlin Zhang[2], Hua Yue[2], Huijuan Liu[1], Baojie Li[1,3,6], Feng Yin [3,4,5,6]

[1]Bio-X Institutes, Key Laboratory for the Genetics of Developmental and Neuropsychiatric Disorders, Ministry of Education, Shanghai Jiao Tong University, Shanghai, 200240, China.

[2]Department of Osteoporosis and Bone Diseases, Shanghai Clinical Research Center of Bone Disease, Shanghai Jiao Tong University Affiliated Sixth People's Hospital, Shanghai 200233, China.

[3]Institute of Traditional Chinese Medicine and Stem Cell Research, Chengdu University of Traditional Chinese Medicine, Sichuan 611137, China.

[4]Translational Medical Center for Stem Cell Therapy, Shanghai East Hospital, Tongji University, Shanghai 200120, China.

[5]Department of Joint and Sports Medicine, East Hospital, Tongji University School of Medicine, Shanghai 200092, China.

[6] Corresponding authors

Baojie Li: libj@sjtu.edu.cn or

Feng Yin: 001yinfeng@sina.com.

This file includes:

Supplementary table 1

Supplementary Figures 1 to 7

Supplementary table 1

**Table:** primer sequences for quantitative PCR	
Runx2	Sense: 5'-CCGGTCTCCTTCCAGGAT-3'<br>Antisense: 5'-GGGAACTGCTGTGGCTTC-3'
Ocn	Sense: 5'-AAGCAGGAGGGCAATAAGGT-3'<br>Antisense: 5'-TTTGTAGGCGGTCTTCAAGC-3'
Col2	Sense: 5'-CCTCAAGGCAAAGTTGGTCCT-3'<br>Antisense: 5'-CACACGTCTCGGTCATGGTA-3'
Sox5	Sense: 5'- CCCGTGATCCAGAGCACTTAC-3'<br>Antisense: 5'- CCGCAATGTGGTTTTCGCT-3'
Cebpα	Sense: 5'-GGACAAGAACAGCAACGAG-3'<br>Antisense: 5'-TCACTGGTCAACTCCAGCAC-3'
Pparγ	Sense: 5'- TCGCTGATGCACTGCCTATG-3'<br>Antisense: 5'- GAGAGGTCCACAGAGCTGATT-3'
Col1a1	Sense 5'- TAAGGGTCCCCAATGGTGAGA -3'<br>Antisense5'-GGGTCCCTCGACTCCTACAT -3'
GAPDH	Sense: 5'-TGACCTCAACTACATGGTCTACA-3'<br>Antisense: 5'-CTTCCCATTCTCGGCCTTG-3'
Runx2: Run-related transcription factor 2; *Ocn*: osteocalcin; *Sox5*: SRY-box transcription factor 5; *Cebpα*: CCAAT/enhancer binding protein alpha; *Pparγ*: peroxisome proliferator-activated receptor gamma; *GAPDH*: glyceraldehyde-3-phosphate dehydrogenase.	

Supplementary Figures

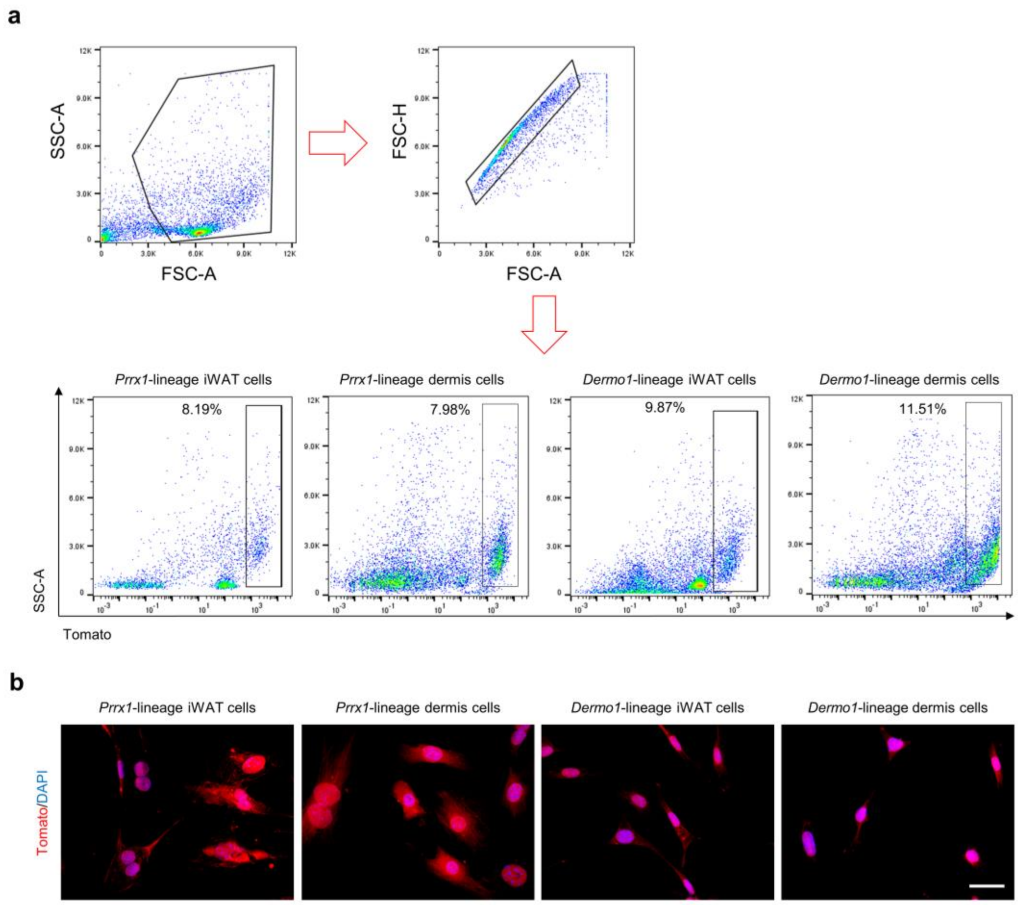

Supplementary Figure 1. Isolation of *Prrx1*- and *Dermo1*-lineage cells from iWAT and dermis.

a. Representative flow cytometry results showing Tomato$^+$ cells in the inguinal white adipose and dermal tissues of *Prrx1-Cre; R26*^{tdTomato} and *Dermo1-Cre; R26*^{tdTomato} mice. FACS gating strategies were shown in upper panels. **b.** FACS-sorted *Prrx1*-lineage iWAT cells, *Prrx1*-lineage dermis cells, *Dermo1*-lineage iWAT cells, and *Dermo1*-lineage dermis cells were Tomato$^+$ in cultures. Scale bars = 20 μm.

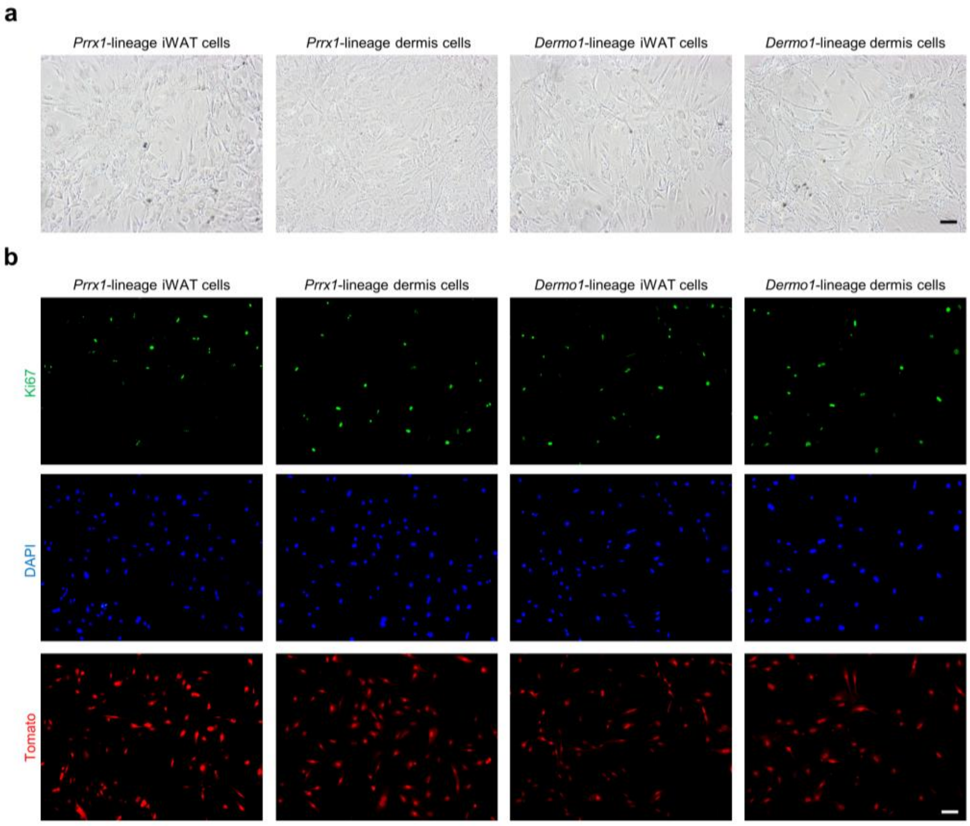

Supplementary Figure 2. The 4 populations of cells adhered to culture plates and underwent proliferation in vitro.

a. The 4 groups of cells adhered to the culture plates. Scale bars = 100 μm. **b.** Immunostaining results for Ki67 and DAPI in the four cell groups (cultured for 5 days). Scale bars= 100 μm. The merged images were shown in main Figure **2a**.

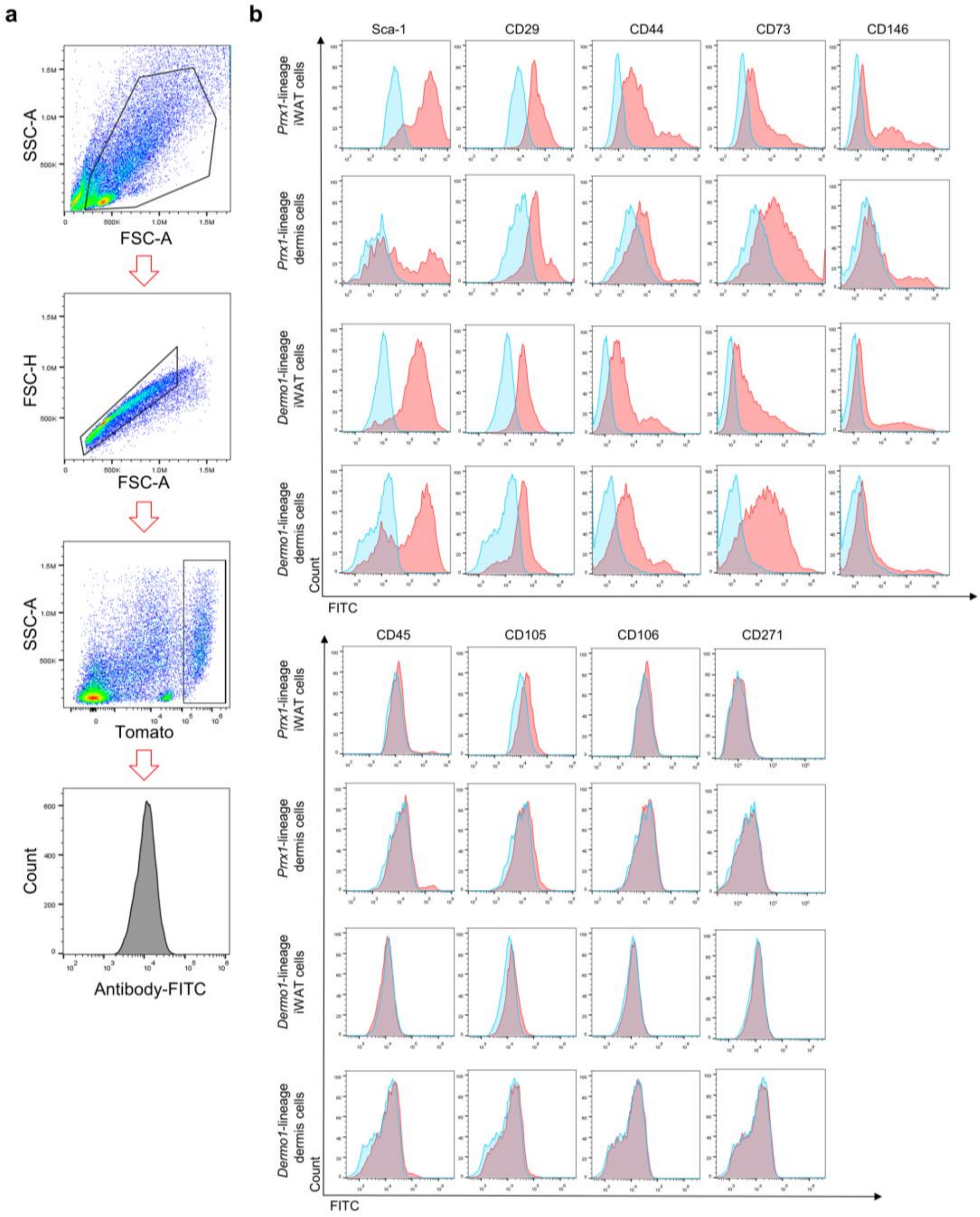

Supplementary Figure 3. Representative results showing the expression of cell surface markers on the 4 groups of cells.

a. The gating strategies for analysis of cell surface markers. **b.** The expression of cell surface markers on the 4 groups of cells.

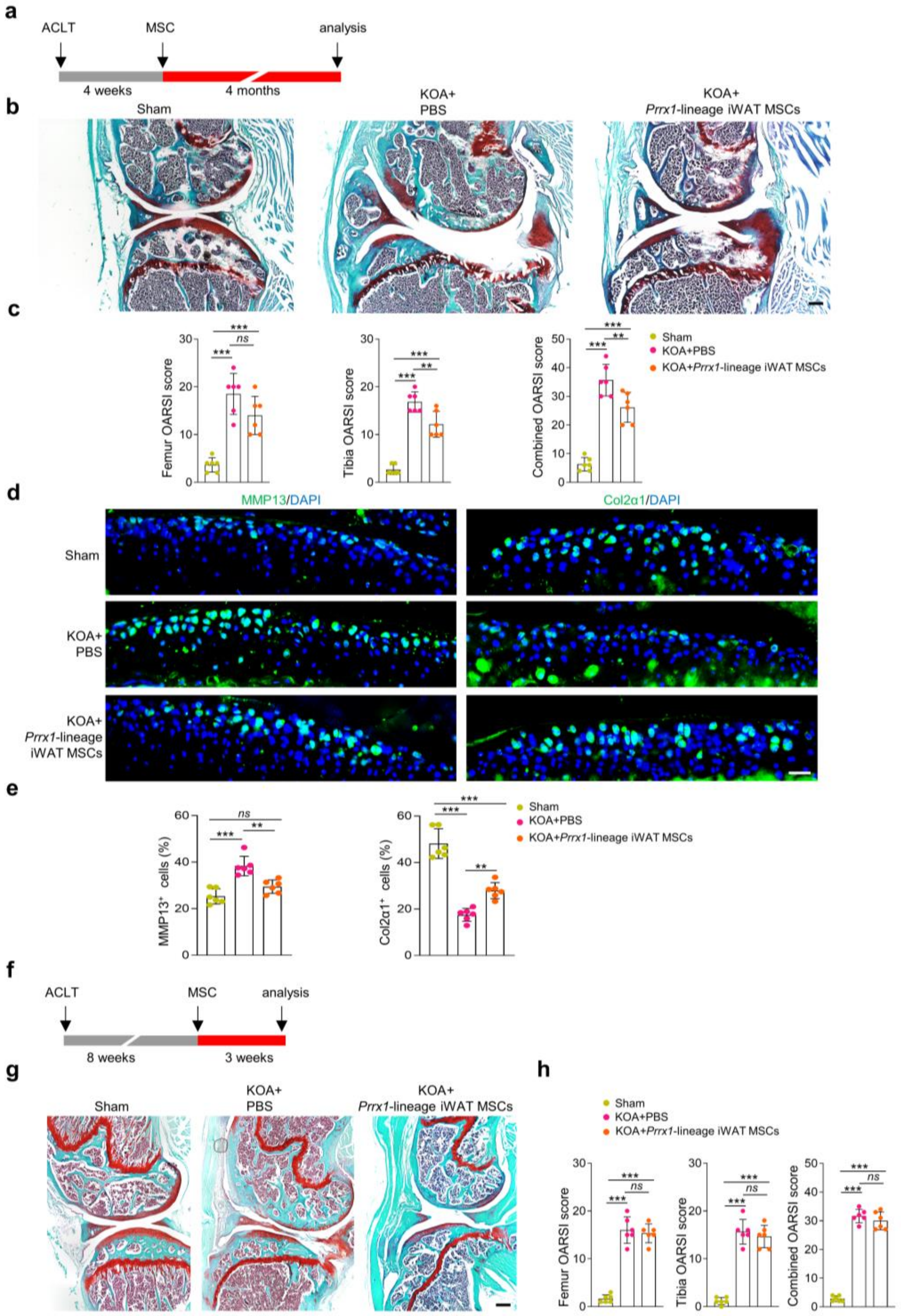

Supplementary Figure 4. *Prrx1*-lineage iWAT MSCs have a long-term therapeutic effect on early stage KOA.

a. Schematic diagram showing the experiment design for **b-e**. **b**. Representative Safranin O staining results of the knee joint articular cartilage. Scale bars = 200 μm. **c.** OARSI scores of the articular cartilage after treatment with MSCs. OARSI scores of femur (left), tibia (middle), and the combined (right). N=6 mice. **d.** Immunostaining for

MMP13 and Col2α1 on the articular cartilage sections. Scale bars = 50 μm. **e.** Quantification results of MMP13 or Col2α1 positive cells. N=6 mice. **f.** Schematic diagram showing the experiment design for **g** and **h**. **g.** Representative Safranin O staining results of the knee joint articular cartilage. Scale bars = 200 μm. **h.** OARSI scores of the articular cartilage after treatment with MSCs. OARSI scores of femur (left), tibia (middle), and the combined (right). N=6 mice. Data are presented as means ±SEM in (**c**, **e**, and **h**). One-way ANOVA (and nonparametric) multiple comparisons was applied in (**c**, **e**, and **h**), $p<0.05$ was considered as statistically significant. *$p < 0.05$, **$p < 0.01$, ***$p < 0.001$, and ****$p < 0.0001$. ns = not significant.

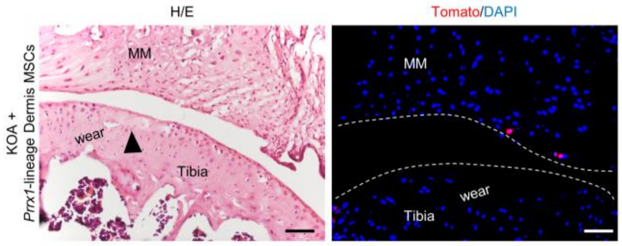

Supplementary Figure 5. Tracing of *Prrx1* dermal MSCs (Tomato$^+$) three weeks after being injected into the intra-articular cavities of OA model mice.

Left panel: H/E staining, right panel: Tomato and DAPI staining. Scale bars = 50 µm. Arrowhead: wear. MM: medial meniscus.

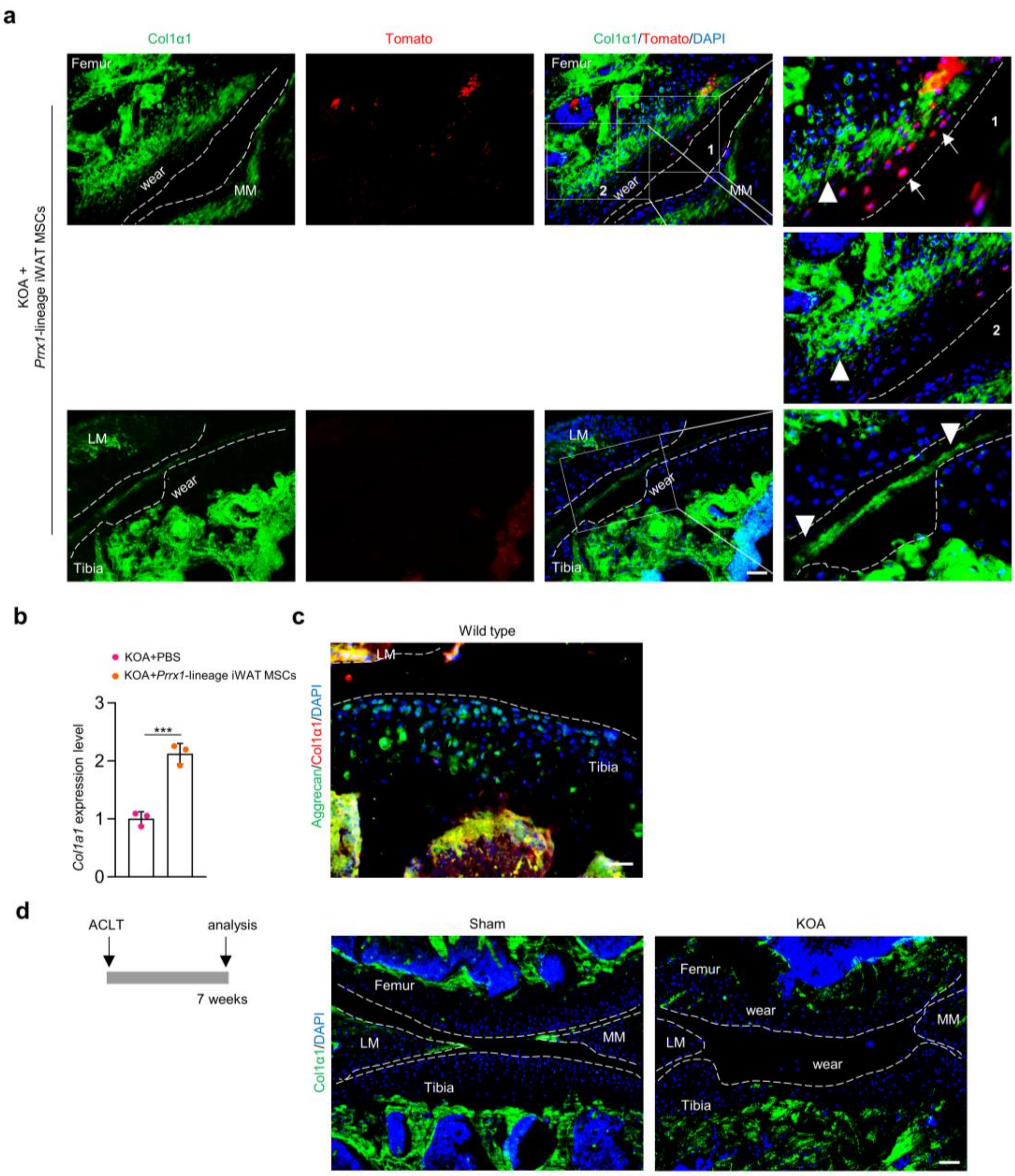

Supplementary Figure 6. ***Prrx1*****-lineage iWAT MSCs induced Col1α1 expression in resident chondrocytes.**

a. Representative immunostaining results for Col1α1 in articular cartilage with (upper panels) or without (lower panels) incorporation of Tomato$^+$ MSCs. Scale bars= 50 μm. **b.** Quantitative PCR was carried out to determine the expression of *Col1a1* in the articular cartilage samples of KOA mice and KOA mice treated with *Prrx1*-lineage iWAT MSCs. N=3 mice. **c.** Normal mice showed none Col1α1$^+$ chondrocytes in the articular cartilage. The joint sections were stained for Aggrecan (a chondrocyte marker) and Col1α1. Scale bars = 50 μm.**d**. Left panel: schematic diagram showing the experiment design. Right panels: immunostaining results for Col1α1 on joint sections

of KOA mice and sham mice. The joint sections were stained for Col1α1 and DAPI. MM: medial meniscus, LM: lateral meniscus. Scale bars = 100 μm. Data are presented as means ±SEM. Unpaired two-tailed Student's t test were applied in **b**, $p<0.05$ was considered as statistically significant. *$p < 0.05$, **$p < 0.01$, ***$p < 0.001$, and ****$p < 0.0001$.

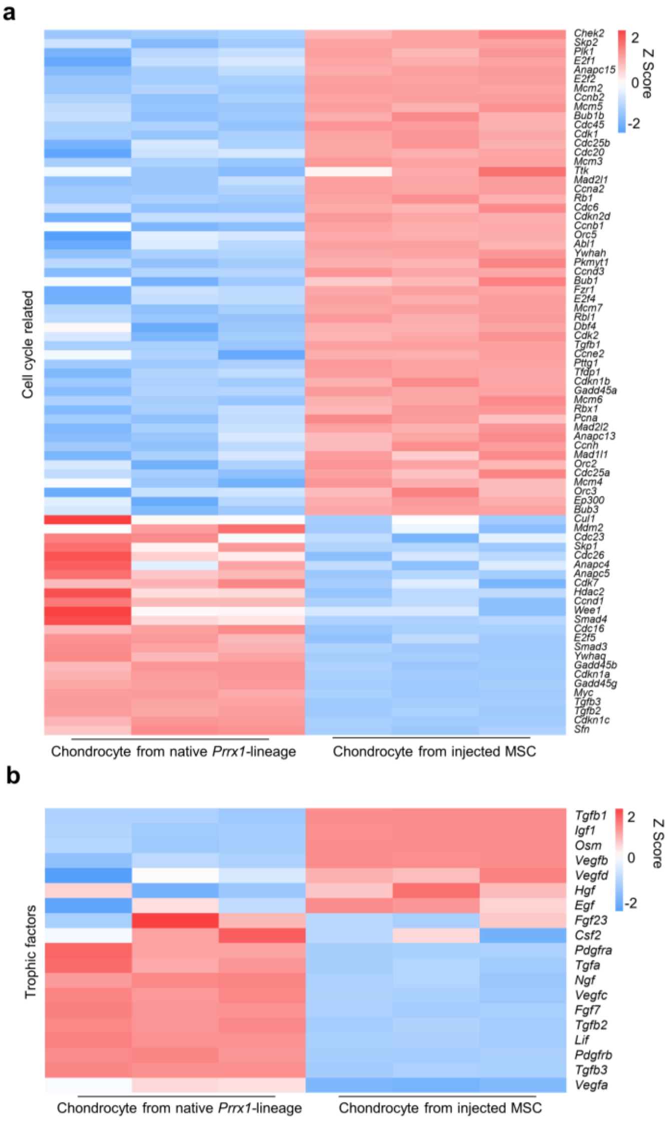

Supplementary Figure 7. Analysis of the transcription profiles of articular chondrocytes derived from *Prrx1*-linege iWAT MSCs.

a. Heatmaps of the cell cycle genes expressed in Tomato^{+} chondrocytes (derived from injected MSCs) and endogenous articular chondrocytes. N=3 mice. **b.** Heatmaps of the trophic factor genes expressed in Tomato^{+} chondrocytes (derived from injected MSCs) and endogenous articular chondrocytes. N=3 mice.
