# Supplementary material for: Comparison studies identify mesenchymal stromal cells with potent regenerative activity in osteoarthritis treatment
Source: NPJ Regen Med. 2024 Apr 1;9:14. doi: 10.1038/s41536-024-00358-y (PMC10984924; doi:10.1038/s41536-024-00358-y)
Supplement: Supplementary file 2 — nr-reporting-summary [file 41536_2024_358_MOESM2_ESM.pdf]

nature portfolio

Corresponding author(s): Baojie Li, Feng Yin

Last updated by author(s): Mar 16, 2024

Reporting Summary

Nature Portfolio wishes to improve the reproducibility of the work that we publish. This form provides structure for consistency and transparency in reporting. For further information on Nature Portfolio policies, see our Editorial Policies and the Editorial Policy Checklist.

Statistics

For all statistical analyses, confirm that the following items are present in the figure legend, table legend, main text, or Methods section.

n/a	Confirmed	
☐	☒	The exact sample size (*n*) for each experimental group/condition, given as a discrete number and unit of measurement
☐	☒	A statement on whether measurements were taken from distinct samples or whether the same sample was measured repeatedly
☐	☒	The statistical test(s) used AND whether they are one- or two-sided<br>*Only common tests should be described solely by name; describe more complex techniques in the Methods section.*
☒	☐	A description of all covariates tested
☒	☐	A description of any assumptions or corrections, such as tests of normality and adjustment for multiple comparisons
☒	☐	A full description of the statistical parameters including central tendency (e.g. means) or other basic estimates (e.g. regression coefficient) AND variation (e.g. standard deviation) or associated estimates of uncertainty (e.g. confidence intervals)
☒	☐	For null hypothesis testing, the test statistic (e.g. *F*, *t*, *r*) with confidence intervals, effect sizes, degrees of freedom and *P* value noted<br>*Give P values as exact values whenever suitable.*
☒	☐	For Bayesian analysis, information on the choice of priors and Markov chain Monte Carlo settings
☒	☐	For hierarchical and complex designs, identification of the appropriate level for tests and full reporting of outcomes
☒	☐	Estimates of effect sizes (e.g. Cohen's *d*, Pearson's *r*), indicating how they were calculated

Our web collection on statistics for biologists contains articles on many of the points above.

Software and code

Policy information about availability of computer code

Data collection	For the RNA-Seq data, raw sequencing data were collected from Illumina Nova Seq 6000 platform.<br>RNA from the barcoded cells was subsequently reverse-transcribed and sequencing libraries constructed with reagents from a Chromium Single Cell 3' v3 reagent kit (10X Genomics) following the manufacturer's instructions. Sequencing was performed with Illumina NovaSeq 6000 (Illumina).
Data analysis	Bulk RNA-Seq data analysis was performed as follows: (1) Data filtering, the raw data obtained from sequencing was filtered using SOAPnuke (v1.5.6) to filter out 1) reads containing adapter (adapter contamination); 2) reads with an unknown base N content greater than 5%, and 3) low-quality reads (reads with a mass value of less than 15 and more than 20% of the total base number of the reads are low-quality reads) to obtain clean data. Follow-up use of Dr. Tom's Multi-Omics Data Mining (https://biosys.bgi.com) Department conducts data analysis, mapping and mining. (2) Differential gene analysis was performed, and the clean data was aligned to the reference genome using HISAT2 (v2.1.0) software. Use Bowtie2 (v2.3.4.3) to align the clean data to the reference gene set. Gene expression quantification was performed using RSEM (v1.3.1) software, and clustering heat maps of gene expression in different samples were plotted using pheatmap (v1.0.8). Differential gene testing was performed using DESeq2 (v1.4.5) [with Q value≤0.05 or FDR≤0.001. (3) KEGG and GO enrichment analysis, Phyper was used to perform GO (http://www.geneontology.org/) and KEGG (https://www.kegg.jp/) enrichment analysis of differential genes, with Qvalue ≤0.05 as the threshold, and the definition of meeting this condition was significant enrichment in candidate genes.<br>Raw reads were demultiplexed and mapped to the mouse reference genome by Cell Ranger version 6.0.2 (10X Genomics) pipeline using default parameters. Each tissue were sequenced at a depth of about 70% saturation. The generated gene-cell expression matrices were used for subsequent analysis in R version 4.3.1 using Seurat version 4.3.0.1. "Cells" fit any of the following criteria were excluded: <200 expressed genes, >20% UMIs mapped to mitochondria. Samples from iWAT or dermis were respectively integrated using "FindIntegrationAnchors" and "IntegrateData" functions. Integrated data were undergone standard cell cycle regression process provided by Seurat. Processed data were

used for downstream graph-based clustering and t-SNE visualization. "FeaturePlot" function in Seurat was used for the visualization of Specific genes' expression.

For manuscripts utilizing custom algorithms or software that are central to the research but not yet described in published literature, software must be made available to editors and reviewers. We strongly encourage code deposition in a community repository (e.g. GitHub). See the Nature Portfolio guidelines for submitting code & software for further information.

Data

Policy information about availability of data

All manuscripts must include a data availability statement. This statement should provide the following information, where applicable:

- Accession codes, unique identifiers, or web links for publicly available datasets
- A description of any restrictions on data availability
- For clinical datasets or third party data, please ensure that the statement adheres to our policy

The datasets in this work were generated or processed with publicly available software. All the details of the analysis codes used in the manuscript are provided in methods

Research involving human participants, their data, or biological material

Policy information about studies with human participants or human data. See also policy information about sex, gender (identity/presentation), and sexual orientation and race, ethnicity and racism.

Reporting on sex and gender	*Use the terms sex (biological attribute) and gender (shaped by social and cultural circumstances) carefully in order to avoid confusing both terms. Indicate if findings apply to only one sex or gender; describe whether sex and gender were considered in study design; whether sex and/or gender was determined based on self-reporting or assigned and methods used. Provide in the source data disaggregated sex and gender data, where this information has been collected, and if consent has been obtained for sharing of individual-level data; provide overall numbers in this Reporting Summary. Please state if this information has not been collected. Report sex- and gender-based analyses where performed, justify reasons for lack of sex- and gender-based analysis.*
Reporting on race, ethnicity, or other socially relevant groupings	*Please specify the socially constructed or socially relevant categorization variable(s) used in your manuscript and explain why they were used. Please note that such variables should not be used as proxies for other socially constructed/relevant variables (for example, race or ethnicity should not be used as a proxy for socioeconomic status). Provide clear definitions of the relevant terms used, how they were provided (by the participants/respondents, the researchers, or third parties), and the method(s) used to classify people into the different categories (e.g. self-report, census or administrative data, social media data, etc.) Please provide details about how you controlled for confounding variables in your analyses.*
Population characteristics	*Describe the covariate-relevant population characteristics of the human research participants (e.g. age, genotypic information, past and current diagnosis and treatment categories). If you filled out the behavioural & social sciences study design questions and have nothing to add here, write "See above."*
Recruitment	*Describe how participants were recruited. Outline any potential self-selection bias or other biases that may be present and how these are likely to impact results.*
Ethics oversight	*Identify the organization(s) that approved the study protocol.*

Note that full information on the approval of the study protocol must also be provided in the manuscript.

Field-specific reporting

Please select the one below that is the best fit for your research. If you are not sure, read the appropriate sections before making your selection.

☒ Life sciences ☐ Behavioural & social sciences ☐ Ecological, evolutionary & environmental sciences

For a reference copy of the document with all sections, see nature.com/documents/nr-reporting-summary-flat.pdf

Life sciences study design

All studies must disclose on these points even when the disclosure is negative.

Sample size	The size of the sample is based on published articles
Data exclusions	No data exclusions
Replication	The experiments were carried out at least three times or at least three animals in each group
Randomization	Experimental groups were defined by experimental conditions. Within an experimental condition, the allocation was random.

Blinding	The experimental and quantification of results were carried out by two independent researchrers

Reporting for specific materials, systems and methods

We require information from authors about some types of materials, experimental systems and methods used in many studies. Here, indicate whether each material, system or method listed is relevant to your study. If you are not sure if a list item applies to your research, read the appropriate section before selecting a response.

Materials & experimental systems

n/a	Involved in the study
☐	☒ Antibodies
☒	☐ Eukaryotic cell lines
☒	☐ Palaeontology and archaeology
☐	☒ Animals and other organisms
☒	☐ Clinical data
☒	☐ Dual use research of concern
☒	☐ Plants

Methods

n/a	Involved in the study
☒	☐ ChIP-seq
☐	☒ Flow cytometry
☒	☐ MRI-based neuroimaging

Antibodies

Antibodies used

FACS antibodies:
Sca-1-FITC (Biolegend, Cat #: 108105, Clone: D7, 1:50), CD29-FITC (Biolegend, Cat #: 102205, Clone: HMβ1-1, 1:50), CD44-APC (Biolegend, Cat #: 103011, Clone: IM7, 1:200), CD45-FITC (Biolegend, Cat #: 103107, Clone: 30-F11, 1:200), CD45-PE594 (Biolegend, Cat #: 103145, Clone: 30-F11, 1:200), CD73-APC (Biolegend, Cat #:127209, Clone: TY/11.8, 1:200), CD146-APC (Biolegend, Cat #: 134711, Clone: ME-9F1, 1:200), CD105-AF488 (Biolegend, Cat #: 120405, Clone: MJ7/18, 1:250), CD106-FITC(Biolegend, Cat #: 105705, Clone: 429(MVCAM.A), 1:200), and CD271-FITC(Biolegend, Cat #: 345103, Clone: ME20.4, 1:100).
Primary antibodies for Immunofluorescence (IF):
polyclonal anti-Col2α1 antibody (abcam, Cat #: ab34712, rabbit, 1:50), anti-MMP13 antibody (abcam, Cat #: ab39012, rabbit, 1:100), anti-Col1α1 antibody (abcam, Cat #: ab21286, rabbit, 1:100), anti-Aggrecan antibody (millipore, Cat #: AB1031, rabbit, 1:50), anti-Col1α1 antibody (proteintech, Cat #: 67288-1, mouse, 1:100), and anti-CD45 antibody (abcam, Cat #: ab10558, rabbit, 1:100).
Secondary antibodies for FACS, Immunofluorescence (IF):
Alexa Fluor IgG(H+L) 488 (invitrogen, Cat #: A11008, goat anti-rabbit, 1:100) or Alexa Fluor IgG(H+L) 555 (invitrogen, Cat #: A11001, goat anti-mouse, 1:100).

Validation

FACS antibodies:
Sca-1-FITC (Biolegend, Cat #: 108105, Clone: D7, 1:50). Cridland SO, et al. 2009. Blood Cell. Mol. Dis. 45:149.
CD29-FITC (Biolegend, Cat #: 102205, Clone: HMβ1-1, 1:50). Baker CM, et al. 2012. PNAS.
CD44-APC (Biolegend, Cat #: 103011, Clone: IM7, 1:200). Kmieciak M, et al. 2009. J. Transl. Med. 7:89.
CD45-FITC (Biolegend, Cat #: 103107, Clone: 30-F11, 1:200). Podd BS, et al. 2006. J. Immunol. 176:6532.
CD45-PE594 (Biolegend, Cat #: 103145, Clone: 30-F11, 1:200). Haynes NM, et al. 2007. J. Immunol. 179:5099.
CD73-APC (Biolegend, Cat #: 127209, Clone: TY/11.8, 1:200). Ben-Moshe S, et al. 2019. Nat Metab. 1:899.
CD146-APC (Biolegend, Cat #: 134711, Clone: ME-9F1, 1:200). Han Y, et al. 2019. J Clin Invest.
CD105-AF488 (Biolegend, Cat #: 120405, Clone: MJ7/18, 1:250). Sanmarco LM, et al. 2021. Nature. 590:473.
CD106-FITC (Biolegend, Cat #: 105705, Clone: 429(MVCAM.A), 1:200). Monnier J, et al. 2012. J. Immunol. 189:956.
CD271-FITC (Biolegend, Cat #: 345103, Clone: ME20.4, 1:100). Baker D, et al. 1989. Cancer Res. 49:4142-4146.
Primary antibodies for Immunofluorescence (IF) (PMID):
polyclonal anti-Col2α1 antibody (abcam, Cat #: ab34712, rabbit, 1:50). PMID: 36263100
anti-MMP13 antibody (abcam, Cat #: ab39012, rabbit, 1:100). PMID: 37692479
anti-Col1α1 antibody (abcam, Cat #: ab21286, rabbit, 1:100). PMID: 36528128
anti-Aggrecan antibody (millipore, Cat #: AB1031, rabbit, 1:50). PMID: 24625978
anti-Col1α1 antibody (proteintech, Cat #: 67288-1, mouse, 1:100). PMID: 32661339
anti-CD45 antibody (abcam, Cat #: ab10558, rabbit, 1:100). PMID: 34890068
Secondary antibodies for FACS, Immunofluorescence (IF) (PMID):
Alexa Fluor IgG(H+L) 488 (invitrogen, Cat #: A11008, goat anti-rabbit, 1:100). PMID: 36085347
Alexa Fluor IgG(H+L) 555 (invitrogen, Cat #: A11001, goat anti-mouse, 1:100). PMID: 36450710

Animals and other research organisms

Policy information about studies involving animals; ARRIVE guidelines recommended for reporting animal research, and Sex and Gender in Research

Laboratory animals	Information in manuscript "Mice maintenance"
Wild animals	Information in manuscript "Mice maintenance"

Reporting on sex | Information in manuscript "Mice maintenance"

Field-collected samples | Information in manuscript "Mice maintenance"

Ethics oversight | Information in manuscript "Ethics approval"

Note that full information on the approval of the study protocol must also be provided in the manuscript.

Plants

Seed stocks | *Report on the source of all seed stocks or other plant material used. If applicable, state the seed stock centre and catalogue number. If plant specimens were collected from the field, describe the collection location, date and sampling procedures.*

Novel plant genotypes | *Describe the methods by which all novel plant genotypes were produced. This includes those generated by transgenic approaches, gene editing, chemical/radiation-based mutagenesis and hybridization. For transgenic lines, describe the transformation method, the number of independent lines analyzed and the generation upon which experiments were performed. For gene-edited lines, describe the editor used, the endogenous sequence targeted for editing, the targeting guide RNA sequence (if applicable) and how the editor was applied.*

Authentication | *Describe any authentication procedures for each seed stock used or novel genotype generated. Describe any experiments used to assess the effect of a mutation and, where applicable, how potential secondary effects (e.g. second site T-DNA insertions, mosiacism, off-target gene editing) were examined.*

Flow Cytometry

Plots

Confirm that:

☒ The axis labels state the marker and fluorochrome used (e.g. CD4-FITC).

☒ The axis scales are clearly visible. Include numbers along axes only for bottom left plot of group (a 'group' is an analysis of identical markers).

☒ All plots are contour plots with outliers or pseudocolor plots.

☒ A numerical value for number of cells or percentage (with statistics) is provided.

Methodology

Sample preparation | Information in manuscript" Methods(Cell flow cytometry and sorting)"

Instrument | All flow cytometry analysis was performed on Beckman Cytoflex S and all flow cytometry sorting was performed on a Bio-Rad S3e

Software | Acquisition: Beckman CytExpert software. Analsyis: Flowjo V10 software

Cell population abundance | Information in manuscript" Methods(Cell flow cytometry and sorting)"

Gating strategy | Information in Supplementary figure 1 and 3

☒ Tick this box to confirm that a figure exemplifying the gating strategy is provided in the Supplementary Information.
